# Group-selection via aggregative propagule-formation enables cooperative multicellularity in an individual based, spatial model

**DOI:** 10.1371/journal.pcbi.1012107

**Published:** 2024-05-07

**Authors:** István Oszoli, István Zachar

**Affiliations:** 1 Department of Plant Systematics, Ecology and Theoretical Biology, Eötvös Loránd University, Budapest, Hungary; 2 HUN-REN Institute of Evolution, Centre for Ecological Research, Budapest, Hungary; Princeton University, UNITED STATES

## Abstract

The emergence of multicellularity is one of the major transitions in evolution that happened multiple times independently. During aggregative multicellularity, genetically potentially unrelated lineages cooperate to form transient multicellular groups. Unlike clonal multicellularity, aggregative multicellular organisms do not rely on kin selection instead other mechanisms maintain cooperation against cheater phenotypes that benefit from cooperators but do not contribute to groups. Spatiality with limited diffusion can facilitate group selection, as interactions among individuals are restricted to local neighbourhoods only. Selection for larger size (e.g. avoiding predation) may facilitate the emergence of aggregation, though it is unknown, whether and how much role such selection played during the evolution of aggregative multicellularity. We have investigated the effect of spatiality and the necessity of predation on the stability of aggregative multicellularity via individual-based modelling on the ecological timescale. We have examined whether aggregation facilitates the survival of cooperators in a temporally heterogeneous environment against cheaters, where only a subset of the population is allowed to periodically colonize a new, resource-rich habitat. Cooperators constitutively produce adhesive molecules to promote aggregation and propagule-formation while cheaters spare this expense to grow faster but cannot aggregate on their own, hence depending on cooperators for long-term survival. We have compared different population-level reproduction modes with and without individual selection (predation) to evaluate the different hypotheses. In a temporally homogeneous environment without propagule-based colonization, cheaters always win. Predation can benefit cooperators, but it is not enough to maintain the necessary cooperator amount in successive dispersals, either randomly or by fragmentation. Aggregation-based propagation however can ensure the adequate ratio of cooperators-to-cheaters in the propagule and is sufficient to do so even without predation. Spatiality combined with temporal heterogeneity helps cooperators via group selection, thus facilitating aggregative multicellularity. External stress selecting for larger size (e.g. predation) may facilitate aggregation, however, according to our results, it is neither necessary nor sufficient for aggregative multicellularity to be maintained when there is effective group-selection.

## Author summary

There is a lot of debate about how and why multicellularity emerged many times independently and what factors contributed to its prevalence. There are many theories why it was advantageous to be multicellular. Factors with direct advantage for aggregation (like avoiding predation) are evident but there are factors with indirect advantages, like spatiality and temporal heterogeneity. The latter can ensure the survival of the cooperative trait through group selection, without kin recognition or selection towards larger size (predation). In our model we investigated this hypothesis. We modelled two types of cells in a temporally heterogenous, spatial environments. Cooperators can associate to form aggregates while cheaters cannot by themselves stick to others but can enjoy the benefits of the aggregate. In resource-rich environments, cooperators have a disadvantage due to slower growth, but only they can create propagules in resource-poor environments. Cheaters therefore need to piggyback propagule-forming cooperators to make it to the next rich habitat. We show that cooperators can survive due to group selection enabled by spatiality in altering environments, without any further mechanism needed.

## Introduction

**Fig 1:**
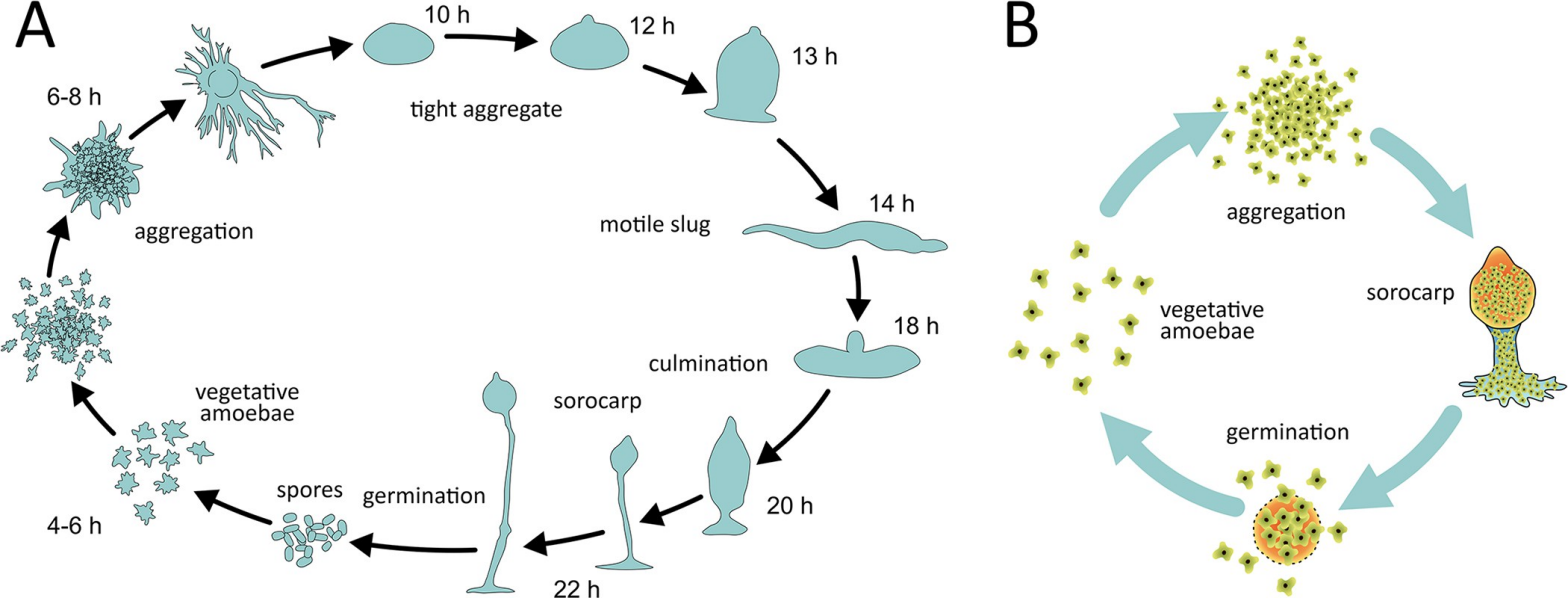
***A***: The life cycle of Dictyostelium discoideum (after *[28])*. ***B***: The simplified life cycle of a generalized aggregative multicellular organism, modelled in this paper. 1. Under normal circumstances cells are free living. 2. During starvation, cells secrete attractive molecules and form aggregates. 3. From the aggregates, fruiting bodies form. In this period there are two cell types: stalk and spore cells. Only spore cells will survive this period, stalk cells die. 4. The spores (or propagules) disperse to the new habitat and cells colonize it. We note that although in reality, cells only aggregate during propagule formation, in our model cooperators constitutively express the adhesive molecules thus can aggregate any time.

The origin of multicellularity is one of the major transitions in evolution [1], arguably the transition that has evolved independently the most, both in prokaryotes [2] and in eukaryotes, at least 25 times [3–12]. This includes both clonal and aggregative types [5,13]. Moderate estimates count only two independent origins of complex (and clonal) multicellularity, all in eukaryotes (Metazoa and Embryophyta) [14,15] (or many more, if Fungi are included [16]). Aggregative multicellularity however is more prevalent and have evolved independently in many protist clades, e.g. in the Discicristata [17], Rhizaria [18], Stramenopiles [19], Alveolata [20] and Holozoa [21] and twice within the Amoebozoa [22], including the model organism *Dictyostelium discoideum*, among others (see [23], Fig 1A).

The common theme in transitions to multicellularity is that independent cells form multicellular groups, and the level of selection shifts from separate individuals to the groups’ level, and (if groups can stably re-appear) a higher level of regulation ultimately evolves to ensure faithful group reproduction and irreversible dependence [13].

In clonal multicellularity, a single cell divides multiple times and the genetically closely related offspring cells stay together (e.g., animals, green plants, various algae, fungi). The more common solution to multicellularity is however the aggregative way of sorocarpic organisms, where unicells aggregate due to an environmental effect (e.g. cAMP starvation signal in *Dictyostelium*) to form a fruiting body (sorocarp) [24,25]. While naturally occurring *D*. *discoideum* fruiting bodies are known to be nearly clonal [26], both laboratory and wild aggregates may consist of unrelated lineages [27], suggesting that chimerism is not a prerequisite for aggregative multicellularity. The life cycle of *D*. *discoideum* is quite complex with several stages after aggregation [28,29]. Ultimately, the aggregated motile slug actively moves to a suitable location to form the fruiting body, in which spores develop, that can spread to colonize new habitats with better life conditions. After sporulation, only cells in the spore may survive and start over as unicells in the new habitat. Those cells who end up in the stalk die, realizing reproductive division of labour–though not based on epigenetic differentiation. As a result, independent strains must cooperate to form a fruiting body with little or no genetic relatedness between cells and different cells compete to gain access into the spore. A generalized and highly simplified aggregative multicellular life cycle that forms the basis of our model, is illustrated by Fig 1B.

The fruiting body of *Dictyostelium* is subject to selection at the group level, and may or may not constitute a higher level evolutionary unit, depending on the amount of information it can stably inherit (according to the definition of [1]). Nevertheless, individual cells are also under selection, and those who can increase their chance of getting into the spore have advantage over other cells, leading to a conflict of interest between individuals (cells must get into the spore to survive) and groups (spores need stalks to ensure effective spread). Aggregative multicellular cycles may have emerged as a way to temporally compartmentalize social conflicts [30].

During emergence of multicellularity (as during any major transition involving the integration of multiple individuals), there must be mechanisms maintaining cooperation between individual cells, otherwise cheaters (with properties advantageous for themselves, but disadvantageous for the group) may win over cooperating cells (with properties advantageous for the group, but disadvantageous for themselves). This is especially true for the aggregative type of multicellularity, as social cheaters are known to emerge both *in silico et vivo* [30–33]. In clonal multicellularity, there is a unicellular bottleneck during the life cycle of the organism [34], ensuring high genetic relatedness of offspring cells, therefore kin selection may ensure the survival of the group (and thus cooperators). However, during the emergence of aggregative multicellularity, kin selection was likely not involved as a cooperation-maintaining mechanism, for multiple reasons. Firstly, aggregative organisms, unlike clonal ones, lack a unicellular bottleneck during their life cycle [34], therefore they cannot keep up the high relatedness between cells. Secondly, kin recognition receptors-like surface protein pairs found in some aggregative multicellular organisms [35,36] are not necessarily efficient at distinguishing kinship at all [37]. Thirdly, there is a trade-off between fast aggregation and specificity of kin recognition [38], that suggests that even if kin recognition would be available, it can be sacrificed for opportunistic reasons. Models have consistently demonstrated that even in case of no active mechanism to positively assort cooperators in groups, like kin recognition or nepotism towards related genotypes, the cooperative trait can survive [31,39–41]. That is, it is enough for cooperators to survive if they have an increased (better than random) chance to participate in groups (which are then better selected), any passive mechanism suffices. Group selection facilitates cooperation, and can manifest via spatial heterogeneity or other forms of population structure (e.g. compartmentation) [42,43]. As cooperators have a higher probability to interact with other cooperators in inhomogeneous populations (compared to the well-mixed case), spatiality facilitates the emergence and maintenance of stable partner relationships [44]. This can be observed at limited spatial diffusion, where cells can only interact with their local neighbourhood where the apparent density of cooperators might be higher than the populational average, hence cooperator groups can form and remain stable against cheaters [44].

Another mechanism to promote cooperation is predation that can effectively shape the behaviour of microbial prey [45]. Cell-to-cell associations may evolve to avoid predation, as associated cells are above the size threshold of the predator thus cannot be preyed upon [46]. Aggregated cells are also more protected from harmful chemicals, such as antibiotics [47–49]. Also, aggregated cells can have higher rate in metabolism [50].

Aggregation assumes an explicitly spatial arrangement of cooperating cells, which in turn entails that cheaters may disrupt the aggregate. Since spatiality facilitates cooperation through increased reciprocity [42,51], it likely had a major impact in at least some origins of aggregative multicellularity. There are of course many other mechanisms that can facilitate (indirect) reciprocation of beneficial acts, leading to cooperation (e.g. kin selection (requires higher level of relatedness), population structure (not a result of spatial structure), reputation (unlikely for microbes), public good production (especially with partner recognition), etc., see [52–55]). However, most models of aggregative multicellularity focus on well-mixed environments. For example [30] examined the connections between motility resource acquisition and group formation in aggregative multicellularity. Arias Del Angel and co. in their review have investigated what effects (generic processes and agent-like behaviours) make distinct aggregative multicellular organisms to behave similarly [56]. A recent study has investigated the development of clonal-like and aggregative-like yeast cells and the competition between the two types [38]. It is common in these studies that they do not separate the effects of spatiality, group selection and predation. A recent model investigated the development of various multicellular life cycles, depending on cell stickiness and the need to avoid predation [57]. While the authors have explicitly modelled regulatory gene networks linking these traits and the status of the environment, their model ignored spatiality (for the parameters and results of their model, see Table A in the S1 Text). Dynamical models of aggregative slime moulds were focused on the effect of variable starvation time on the ecology of strategies [32], selection between different life cycles [58], or on the origin of the aggregative strategy as a first step towards multicellularity [59,60].

We were curious about whether and to what extent can spatiality, individual-level selection (e.g. predation) and group selection facilitate the emergence of aggregative multicellularity and whether any of them is sufficient by itself to achieve the same effect as the unicellular bottleneck and kin selection present in clonal multicellularity. Is a predation bias toward aggregation necessary or can aggregative multicellularity emerge without it? We have constructed an individual based, spatially explicit ecological model to investigate whether spatiality, size-dependent selection, group selection and periodical resource scarcity facilitate the survival of the group-forming (cooperative) phenotypes when cheater (defector) phenotypes are present in the population (assuming unrelated lineages, i.e. omitting kin-selection). For sake of simplicity, we limited our scope on these two ecotypes and to the ecological timescale, ignoring mutation and the evolution of traits, focusing on the ecological stability of the cooperator over different propagation mechanisms. We focused on the ecological timescale to identify whether there is inherent selective advantage provided by aggregation that positively affects the survival of cooperators under predation and/or over multiple colonization events. We tested the hypothesis, whether group selection alone is capable of maintaining the cooperative group-forming behaviour or the more direct benefit of increasing size to reduce predation (or equivalent benefit of size) is necessary.

We assumed that individual cells are free-living, actively moving, and may produce adhesive molecules that enable them to unselectively associate with (and dissociate from) other cells at all times. We impose a variable environment on the population, whereby food is exhausted without replenishment and cells must colonize a new, resource-rich habitat to survive (cf. [32]). When resource becomes scarce, one of the largest aggregates may form a fruiting body with a single propagule containing multiple cells. For sake of simplicity and generality, we ignored most of the biological complexity of *D*. *Discoideum* life cycle in our model organism, assuming a non-motile aggregate. The propagule then disperses to colonize a new, resource-rich and cell-free habitat, effectively sampling the parental aggregate. We were interested in whether this type of simple, non-selective, non-cue-based aggregative behaviour can stably maintain the aggregative phenotype over time against the cheater ecotype, not expressing adhesives. These simplifications allowed us to focus on group selection, excluding genetic effects (e.g. crossing-over, relatedness between individuals, etc.). Alternatively, we have investigated the effect of dispersion by different random fragmentation (independent of aggregate size) and by dispersion, i.e., when the colonizing subpopulation is taken entirely randomly.

## Results

**Fig 2:**
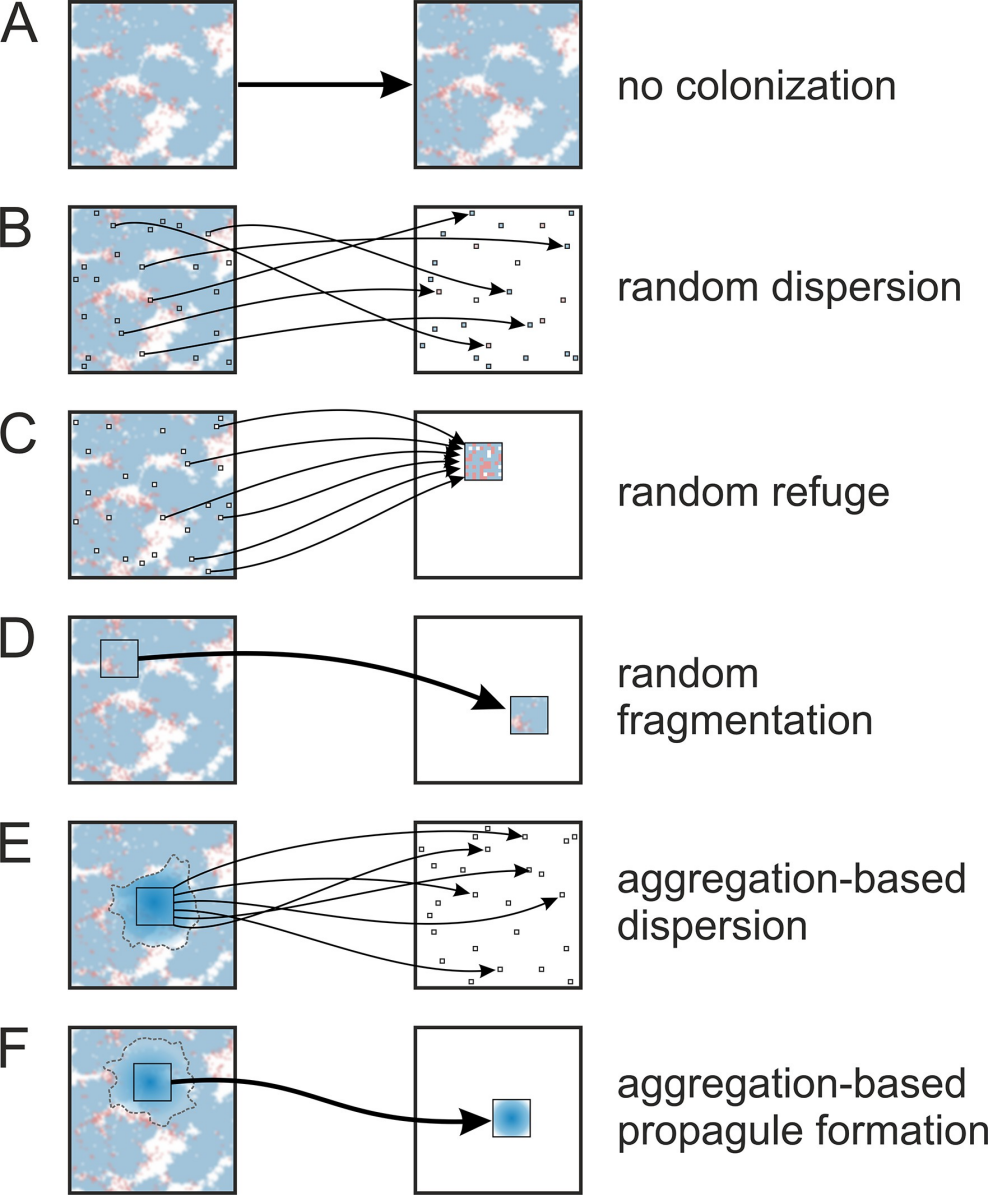
Possible reproduction mechanisms considered for the population when a new habitat is colonized. During selection for colonization (transfer), cells are either selected randomly (B, C, D) or based on strongest aggregate (E, F). After transfer to the new habitat, the population either retains its original structure (D, F) or is dispersed (B, E). For more details, see text. The labels A-F are used consistently in all figures.

In our experiments, we compared five distinct colonization mechanisms (and lack of) during resource scarcity, and the effect of size-dependent selection. The colonization mechanisms define how a subpart of the population is transferred to an empty, new habitat after the old habitat is exhausted. Aggregating cells may either form a propagule, a fragment or disperse without maintaining population structure to the new habitat. The different propagation mechanisms are as follows (labels are used consistently in all figures, including supplementary figures in S1 Text). For an illustration of these mechanisms, see Fig 2.

- A: **No colonization**: Organisms that do not migrate or colonize new habitats at all. Without resupply of the resource, the population will ultimately die out.
- B: **Random dispersion**: Random sampling from the whole habitat, and colonization without a compact propagule, corresponding to bacteria (or single-cell spores) randomly dispersed via air/water to new habitats, or individuals actively and randomly migrating to new habitats.
- C: **Random refuge**: Represents an intermediate stage between B and F, where cells disperse to the new habitat randomly, but form an “aggregate” there, subject to stronger competition. While aggregation does not play a role here, we can investigate the effect of immediate competition of a dense founder population in the new habitat, without retaining its original spatial structure. For example, organisms trapped in drying ponds in the tidal zone of water bodies.
- D: **Random fragmentation**: Random sampling a continuous subset from the spatial population, maintaining structure when transferred to the new habitat, similar to the reproduction of hydra or lichen thalli. Aggregation does not play a role, but the original spatial structure is maintained, and competition is strong in the new habitat due to the compact founding fragment.
- E: **Aggregation-based dispersion**: Represents an intermediate stage between B and F, where aggregates form and one is selected for propagation, but its cells are immediately dispersed in the new habitat. No known biological organism corresponds to this (perhaps multicellular propagules that burst on arrival), but it represents an important intermediate for comparison.
- F: **Aggregation-based propagule formation**: Multicellular aggregations form, are selected for propagation and colonize the new habitat maintaining internal structure. The textbook example is *D*. *Discoideum*, though here we use a simplified model.

The different propagation mechanisms allow varying degrees of competition and conservation of spatial structure (local interactions). Random dispersion (B) does not keep population structure at all (neither during selection for transfer nor on arrival), while aggregation-based colonization fully retains the local population structure of the aggregate, resulting in higher level of competition on arrival (a disadvantage for any cell). C, D and E represent intermediate mechanisms between B and F.

Besides the effect of group selection, we have included selection on the individuals too, in the form of predation, assuming a nondescript filter-feeder microbial species, without saturation limit. Predation affects non-associated cells more, but in general, this effect comes from the fact that cooperators, forming aggregates, effectively increase their size above that of their predators. Thus, smaller individuals pay higher cost of being preyed on, and it becomes beneficial to increase size.

### The effects of group selection and individual selection

**Fig 3:**
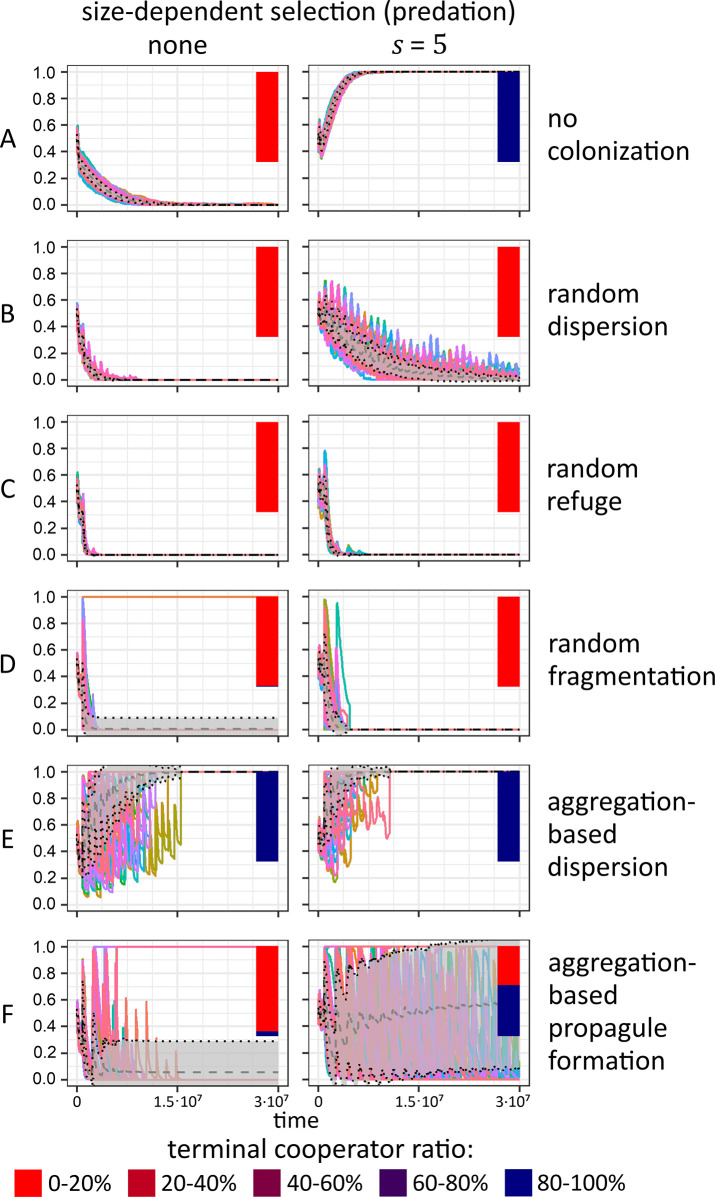
Relative frequency change of cell types over multiple colonization events depending on propagation mechanism (rows) and size-dependent selection (i.e. predation; columns). Each subplot shows the result of 140 independent simulations (coloured lines) with mean (dashed) and ± standard deviation enveloped in grey. The *y* axis shows the ratio of cooperators. Bars on the right of each subplot show the average outcomes categorized into five categories (see legend) by the terminal percentage of cooperators (for more details, see Figs E, F in S1 Text). There is no real coexistence: simulations converge either to a monomorphic cooperator or defector population. If there is no size dependent selection (left column), cooperators can only survive under aggregation-based dispersion (E left) or, with a small probability, under aggregation-based propagule formation (F left). Aggregation-based dispersion is indifferent to size-dependent selection (E right), however, cooperators now can survive with high probability in case of no colonization (A right) or aggregation-based propagule formation (F right). The parameters of these plots are as in Table 1.

When there is no size-dependent selection (Fig 3 left column), only defectors survive in the homogeneous environment (no colonization; Fig 3A left) or when propagation type is random dispersion (Fig 3B left) or random refugium (Fig 3C left). In case of random fragmentation (Fig 3D left) and aggregative propagule formation (Fig 3F left), cooperators survive with a low chance (<5%), clearly the effect of maintaining local interactions between cooperators as the population structure is not disrupted during transfer to the new habitat. In the case of aggregation-based dispersion, cooperators always survive (Fig 3E left), as in this case they enjoy the double benefit of having their aggregate structure maintained during transfer and minimizing initial competition between colonists.

When predation is in effect (Fig 3 right column), the only difference is when there is no colonization (Fig 3A right), and when aggregation-based propagation happens (Fig 3F right). In both cases, size-dependent selection (e.g. predation stress) affects defectors negatively more than cooperators, as defectors are less likely to be in aggregates and are thus subject to stress (predation), giving thus cooperators a huge advantage.

To check that our simulations do not differ significantly due to different growth dynamics of the phenotypes, we have measured both the average number of cell divisions and the elapsed time between successive colonization events in case of the different propagation mechanisms and with or without size-dependent selection. During the allotted simulation times, a different number of colonization events happens under different colonization mechanisms. In cases where cooperators go extinct, there will be no more colonization events, obviously. Thus, colonization mechanisms that cannot maintain cooperators are doomed. This explains the different average total number of colonization events. However, while colonization mechanisms may differ significantly, there is no significant difference in the order of magnitude in either cell division count or elapsed time between successive colonization events depending on the colonization mechanism. In other words, the *rate of colonization* is roughly the same under any colonization mechanism (unlike in [32], where variable replenishment times were studied). On average, 2.03 (0.34 SD) colonization events happened in case of random fragmentation, 11.01 (4.23SD) in case of aggregation-based propagule formation, 2.26 (0.52 SD) in case of refugium and 21.29 (6.14 SD) in case of random dispersion. For details, see Supplementary Material sections 1, 2 in S1 Text.

To investigate the effect of the composition of the initial population on our ecological timescale, and whether there is considerable cooperator threshold effect we ran multiple simulations with different initial cooperator under predation stress ratios (see Figs G, H in S1 Text). As one would expect, increasing the initial ratio of cooperators increases their ultimate survival rate. There is a threshold effect at different values based on colonization mechanism, but we only found a considerable effect in the case of random fragmentation (Fig G(D) in S1 Text) and aggregation-based propagule formation (Fig G(F) in S1 Text), where the threshold is not at the theoretical minimum or maximum of cooperator count. Despite the threshold, it is clear, that aggregation-based propagule formation robustly helps the survival of cooperators.

### The effects of association and dissociation rates

**Fig 4:**
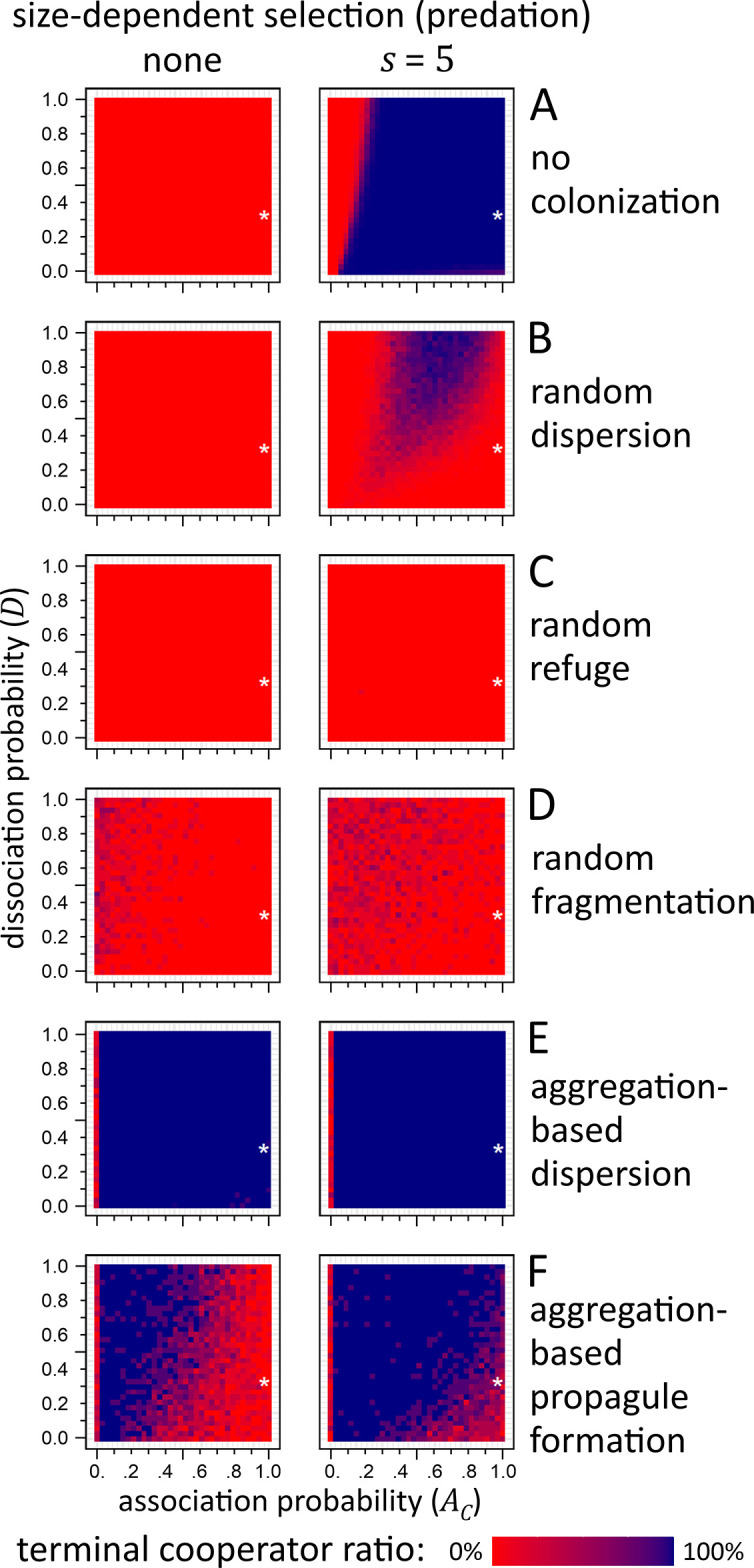
Terminal cooperator ratio depending on association propbability *A*_*C*_ (*x* axis), dissociation probability (*D*_*C*_, y axis), propagation mechanism (rows) and size-dependent selection (*s*, columns; e.g. predation). Each pixel on the heatmap represents the mean of terminal cooperator ratio of 7 independent simulations. The white asterisk denotes the parameters of the first experiment (Fig 3). When size-dependent selection was not in effect (A, left column), only defectors survive in case of no colonization, and the introduction of selection (e.g. predation stress) clearly benefits cooperators with reasonable association rates (A, right column). Random dispersal (B left) shows a slight benefit for cooperators due to decreased competition at the new habitat, but only for high association rate and even higher dissociation rates. In case of random refuge and fragmentation (C, D), size-dependent selection barely has any effect. Aggregation-based dispersion (E) always benefits cooperators, regardless of predation stress or even association/dissociation rates. Aggregation-based propagation is beneficial when there is no predation stress (F left) but is even more advantageous when predation is introduced (F right). For more details and explanation, see text; for the time-evolution of independent simulations for selected (*A*_*C*_, *D*) pairs, see Fig I in S1 Text; for a detailed examination of differences in division at certain (*A*_*C*_, *D*) pairs, see Fig J in S1 Text. Parameters are as in Table *1*, except for aggregation-based fragmentation, where *T* = 150 000 000.

Size dependent selection affects dissociated cells more than associated cells, as free-moving cells are more exposed to environmental stress, for example predators. Since most of the associated cells are cooperators and dissociated cells are mostly defectors, cooperators enjoy a relative fitness advantage compared to defectors. To investigate how much the advantage of cooperators is the result of predation or aggregation-based propagation, we separated the direct benefit of association from the indirect benefit of propagule formation. Accordingly, we examined the effect of predation when there was no propagule formation (i.e., population was destined to ultimately die out due to resource exhaustion) and the effect of propagule formation when there was no predation. Cooperators and defectors strategically differ in their production (or not) of adhesive molecules: as a result, cooperators associate with probability *A*_*C*_, while defectors do not associate (*A*_*D*_ = 0). Cooperators also pay larger metabolic costs due to adhesive production than defectors (*c*_*C*_>*c*_*D*_). They are identical in every other aspect, e.g. share the same dissociation rate *D*, division probability *S*, energy threshold for division *e*, etc. Setting the metabolic cost to any arbitrary value (ensuring cooperators have the disadvantage) means that the only other parameter in which the two types differ is the association rate *A*_*C*_ (for actual parameter values, see Table 1). Fig 4 shows the result of the experiment, with or without predation, depending on different association and dissociation rates. For a detailed examination of the divisional differences between associated and dissociated cells for some of the interesting patterns, seeFig J in S1 Text.

**Table 1:** The symbols, parameters and their values used throughout the paper. Parameters defined separately for cooperators and defectors are denoted with a C or D in subscript, respectively.

	Parameter name	Symbol	Default value	Unit of measurement
Simulation properties	Length of the simulation	*T*	300 000 000	update
	Lattice edge size	*M*	100	cell
	Propagule size	*N*_*P*_	100	individual
	Initial resource value of lattice cells	*r*_0_	100	resource unit
	Minimal total resource amount for the good period	*R*	400 000	resource unit
	Predation strength	*s*	{3, 4, 5, 6, 7}	exponent
	Probability of death due to size dependent selection	*P*	$P=\frac{1}{{\left(N_{a}+2\right)}^{s}}$	probability
Individual attributes	Initial energy value of individuals	*e*_0_	100	resource unit
	Metabolic constant	*c*	*c*_*C*_ = 10, *c*_*D*_ = 8	resource unit
	Energy barrier of reproduction	*d*	190	resource unit
	Probability of cell division	*S*	0.7	probability
	Probability of overwriting another cell	*O*	0.3	probability
	Energetic cost of movement	*m*	0.875	resource unit
	Probability of association	*A*	*A*_*C*_ = 0.95, *A*_*D*_ = 0	probability
	Probability of dissociation	*D*	*D* = 0.3	probability
	Number of associated neighbors	*N*_*a*_	N/A	individual

**Without colonization** (Fig 4A, assuming periodic resource renewal within the same habitat), aggregative cooperators could only survive against defectors if there is predation, as associations provide an advantage for cooperators ensuring that the investment into adhesives (and reduced growth due to higher metabolic cost) is ultimately returned (Fig 4A right). The benefit of cooperation in this case is clearly due to spatial aggregation (requiring moderate levels of *A*_*C*_, but even for cases where *A*_*C*_<*D*) against predators and survival solely depends on cooperator association rate, as aggregated cells are less (or not at all) exposed for predators. Without size dependent selection (Fig 4A left), cooperators ultimately die out, because defectors only have benefits (faster growth) without the truncating selection of predation (or equivalent stress) while cooperators still had to pay the cost of producing adhesives (slower growth).

During **random dispersal** (Fig 4B), the survival of cooperators is confined to a region where *D*>*A*_*C*_ and *A*_*C*_ is not too small. When association ratio is too small, cooperators cannot enjoy the increased size of aggregation against predators. When association rate is higher than dissociation rate, strong aggregation prevents cooperators to spread effectively, overwriting their own type during division with an associated layer of defectors on the perimeter of the aggregate. Cooperators will subsequently be underrepresented during sampling, and as a result, in the new habitat too, leading to their ultimate extinction.

In case of **random refugium**, predation does not help cooperators (Fig 4C right), because the population structure is not maintained during transfer, and it is less likely that 1) cooperators are included and 2) cooperators meet cooperators to quickly form stress-resistant aggregates.

**Random fragmentation** (Fig 4D) removes the benefit of cooperators under predation stress, as it ignores aggregates, disrupting associations with every colonization event. Though this effect is not as pronounced as in case of dispersal, it still exposes previously safely associated cooperators to predators as the transferred fragments are comparably smaller than aggregates (Fig 4D, right). The cause is twofold: cooperators grow and divided slower and, in the average, are less motile than defectors due to being aggregated, hence they are less likely to be included in any random (but continuous) sample of the population. As a result, it is less probable that cooperators are included in a random fragment than in case of propagule formation, decreasing their founding frequency in the new habitat. Only for large association rates could cooperators ensure that 1) they are present in a random fragment in sufficient numbers for transfer, and that 2) they survive against better competing defectors in the new habitat due to larger initial numbers and faster aggregation. In other words, predation is not strong enough to counter-balance the faster growth of defectors, so cooperators are densely surrounded by defectors all around, which are better competitors in the short run. With or without predation, cooperators may have a slightly increased survival chance compared to the no colonization or random dispersal due to the fact that aggregates are more likely to fall into the random square that is transferred.

The best for cooperator survival is **aggregation-based dispersion** (Fig 4E), which always benefits cooperators, regardless of size-dependent selection, association, or dissociation rates. The stable and robust survival of cooperators comes from the fact that this colonization mechanism always favours aggregated cells (which are mostly cooperators) plus in the new habitat the initial competition is low thanks to the distance of dispersed cells. Even predation cannot reduce the advantage of being a competitor.

**Aggregation-based propagule formation** (Fig 4F) maintains the group-selection benefit of cooperators. Cooperators robustly survive even if there is no predation at all (Fig 4F left), while under predation, the benefit of cooperation is obviously even more pronounced (Fig 4F right). The column corresponding to *A*_*C*_ = 0 in Fig 4E and 4F is red because there are no aggregates to choose from and the transfer square is randomly positioned, benefiting defectors (effectively corresponding to random fragmentation).

**Fig 5:**
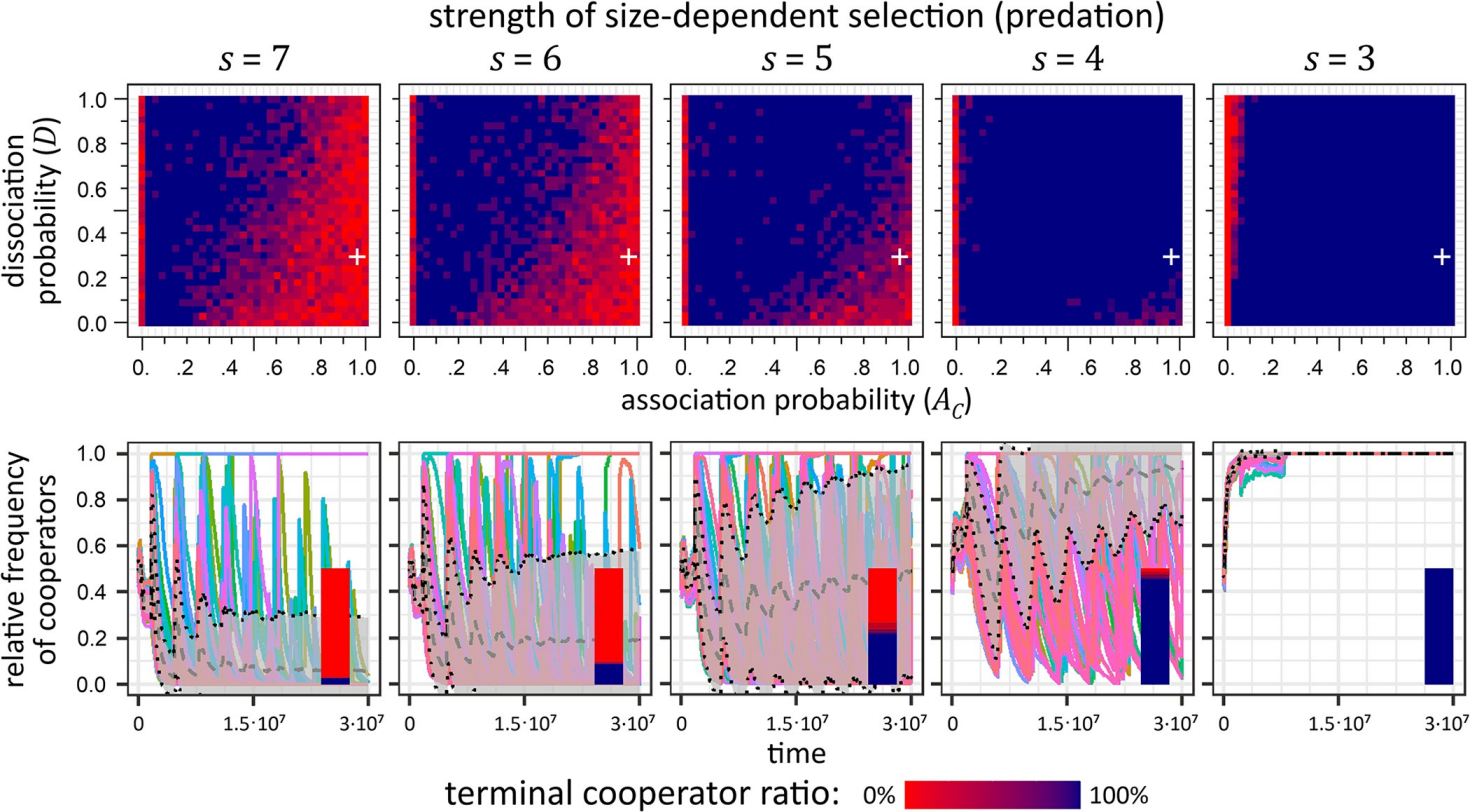
The effect of size-dependent selection (predation) on aggregation-based propagule formation. Top row: terminal cooperator ratio, depending on various association (*A*_*C*_, *x* axis) and dissociation probabilities (*D*, *y* axis); bottom row: temporal dynamics of independent simulations at *A*_*C*_ = 0.95, *D* = 0.3 (indicated by the white crosshair in the top row). First column (*s* = 7) corresponds to weakest, last column (*s* = 3) to strongest predation, cf. Eq 1. The middle case *s* = 5 is the same as in Fig 4F, right panel. Defectors suffer more from predation, because they are less associated, hence as the predation stress increases, the area where they win reduces considerably. As predation decreases, defectors do not suffer from predation load, and cooperators lose their advantage, especially when the association rate is larger than the dissociation rate (*A*_*C*_>*D*). If predation is effectively removed (*s*>7, also Fig 4F, left panel), cooperators can still survive at *A*_*C*_<*D*. For more details, see text and Figs E, F in S1 Text.

We have further explored the effect of the strength of size-dependent selection (predation) on the successful colonization of cooperators to see effect of limited predation. We have explored predation strength values *s* = {3, 4,…,7} according to Eq 1. The effect of different predation strength values in case of aggregation-based propagule formation is illustrated by Fig 5, for all colonization mechanisms, see Figs E, F in S1 Text. The default value (Fig 5 middle, as in Figs 3 and 4) is *s* = 5, in which case the effect of predation is not negligible, but cooperators has a clear advantage over to defectors. When there is no predation, the benefit of cooperators is clearly due to aggregation-based propagation: group-selection and maintenance of aggregate patterns during transfer ensures the long-term stability of cooperator-only neighbourhoods. Decreasing the strength of predation benefits the defectors in case of all colonization mechanisms, as one would expect, although this effect is more reduced in case of aggregation-based dispersion (Fig E(E) in S1 Text), where there is minimal (or no) initial competition after colonization. This gives more chance for cooperators to survive the initial struggle after colonization, while also reducing the effect of starvation as dense aggregates form more slowly (more prevalent in aggregation-based propagule formation Figs 5, Fig E(F) in S1 Text).

**Fig 6:**
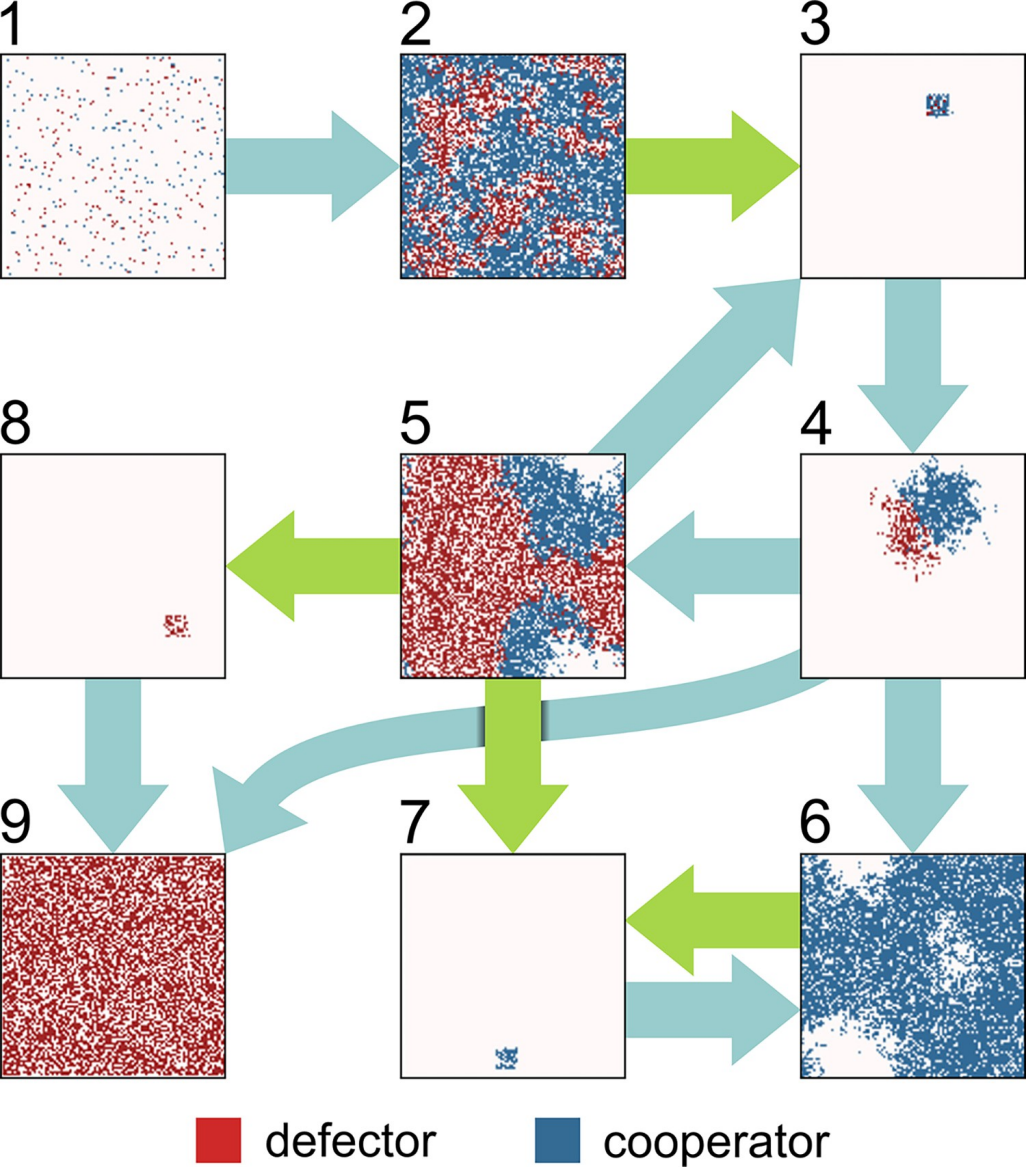
Possible representative states of the simulated population of cooperators (blue) and defectors (red) on lattice. Teal arrows indicate population dynamics, green arrows subpopulation transfer to new habitat. Simulation starts from a random state with low cell count (1). Approximately 4% of the available space is occupied by cells. Cells consume resources, grow in numbers and spread on the lattice (2). After resource is depleted, a propagule is formed, possibly containing both types, and colonizes a new habitat (3) where it starts to spread (4). There are three possibilities: neither type outcompetes the other (5), cooperators outcompete defectors (6), and defectors outcompete cooperators (9). In mixed populations (5), three propagule types can emerge and colonize new habitats: mixed (3), cooperator-only (7) and defector-only (8). Uniform defector populations (9) cannot form propagules and colonize new habitats–without constant resource inflow, the population is doomed. All-cooperator populations (7) can successfully form fruiting bodies and colonize new habitats for eternity, whenever resources are exhausted (6), but without mutation, cheaters will never reappear, and simulation can be terminated.

Interestingly, without predation, propagule formation cannot maintain cooperators at high association rates (*A*_*C*_>*D*, Fig 5 left). This can be explained by the fact that the high density of associated cooperators prevents their spread and increases internal competition (dividing cells overwriting each other), ultimately leading to starvation in the core of aggregates, see corresponding ring structures empty cores in Fig 6, insets 5, 6.

## Discussion

Aggregative multicellularity may mean the first step toward a major evolutionary transition where non-clonal cells cooperate to survive [13]. The maintenance of cooperation in aggregative scenarios poses a challenge, as defectors, cells that do not invest into group-formation but benefit from it, can exploit the public good of an aggregate (e.g., protection against predation). Defectors, therefore, if all else equals, have higher fitness than cooperative cells as they save the cost of producing adhesives and can allocate more resource to opportunistic growth. Besides other mechanisms, group selection is well known to be able to maintain cooperation against cheaters [39,42,43,54]. Spatiality can facilitate cooperation, because individuals can only interact with their neighbours, and cooperators may get a locally larger benefit than they would receive based on average population densities. As a result, cooperator groups can be formed [44]. Since spatiality facilitates cooperation, it must have had a major impact on the emergence of aggregative multicellularity.

All aggregative multicellular organisms are simple and facultative [34]. Simple, because all cells are in direct contact with the external environment at least when their metabolism is active, they connect by producing adhesive molecules, have very limited communication and the resource transfer between cells, lack dedicated cell-to-cell communication methods, genetically encoded developmental program and tissues (as complex multicellular organisms do [61]). They are facultative, because they only become multicellular on an external signal, otherwise they maintain an active single-celled lifestyle [62]. There are likely different mechanisms that prevent aggregative organisms to achieve complex multicellularity.

Firstly, aggregation is more advantageous than clonal development in terms of rapid group formation. Aggregative multicellular organisms tend to live in environments where there is a selection for rapid switch between unicellular and multicellular development, like fruiting body formation during starvation [24], potentially because resources tend to get exhausted within the organisms’ lifetime. Because of the need for fast aggregation and the trade-off between fast aggregation and accuracy of kin recognition, there is always a persistent evolutionary conflict that limits the potential to become complex [38].

Secondly, spending most of their life cycle in unicellular state, aggregative organisms are subject to different selective forces in unicell and in aggregated phases, mutations having different fitness effects. Selection mostly acts on unicellular traits, which makes the fixation of group-beneficial but individually costly mutations difficult or downright impossible [63]. Such mutations could increase group cohesion and evolutionary stability of multicellularity, because they constrain the reversion to unicellularity [64–66], but the truncating selection at the unicell phase acts as a barrier to evolve complex multicellularity [34].

Thirdly, while there is limited between-cell selection in clonal organisms, there is strong competition in aggregative multicellular ones, depending on the length of the unicellular period [27,67]. This likely adds to the factor of strong selection against deviating from the opportunistic fast-growing strategy during resource rich times.

Fourthly, aggregative multicellular organisms are selected to flexibly and optimally switching between growth (unicellular) and survival (multicellular) depending on resource scarcity [34]. This selection towards flexibility limits the route to complexity, as that would mean a commitment to a suboptimal, obligately multicellular state. Besides, obligate multicellular organisms must find multicellular solutions to novel challenges (e.g., different circulatory systems for the diffusion problem in plants, fungi and animals [61]). These adaptations require genetically encoded development, complex mechanisms of cellular signalling, i.e. differentiation, maintenance and repair of the multicellular state [68–70]–mostly not available in aggregative multicellular species.

It is worth to note that obligate aggregative multicellularity is possible theoretically [34]. It requires that the multicellular phase is constitutive, i.e., genetically fixed instead of depending on environmental cues. Researchers have successfully engineered yeast cells to constitutively express the adhesive glycoprotein FLO1 to enforce a continuous multicellular phase of potentially unrelated strains [38,71].

Here, we have modelled a general microbial organism capable of aggregation by motile, unrelated single-cells, akin to *Dictyostelium*, though its lifecycle was greatly simplified to be able to separate the effect of spatiality and propagation mechanism on the survival of cooperators, without modelling all biological particularities. We started our investigation with the most trivial case, and advanced toward more complicated ones to better understand underlying mechanisms. We deliberately set parameter values to give a disadvantage to cooperators in the short run, implementing thus a worst-case scenario. If cooperators can survive despite being worse competitors (compared to defectors), their survival is robust in a larger parameter region. Non-aggregating cells benefit in the short term but are doomed in prolonged starvation in a fluctuating environment, while cooperators may survive for longer and find new resource patches easier. When the environment is unpredictable, both types may coexist [32,72].

We have investigated whether spatiality plays a role in enabling or facilitating the establishment of aggregative multicellularity. We have compared different propagation types that enable colonization of new habitats to the non-propagating population: random fragmentation, aggregation-based propagule formation, refugium and random dispersion. We found significant differences between the mechanisms of colonization in the number of survived cooperators and the number of colonization events. This means that the cells were more successful in the case of aggregation-based propagule formation because they could colonize more habitat and had more offspring on average.

We have also investigated the effect of predation (or equivalent size-dependent selection), where small prey (mostly individual, free-living defector cells) is preyed on, while cooperators can escape predation by forming aggregates. There are many other mechanisms then aggregation, similar in effect, selecting for larger size, and there are many other benefits of increased size, e.g. becoming better predators themselves (e.g. group feeding in the aggregating proteobacteria [73]), improved nutrient intake [50], better protection against harmful molecules [47–49], ability to migrate larger distances [74], etc. This is especially true for aggregations that may be formed of distantly related individuals as seen in schooling fish or merging of *D*. *Discoideum* slugs even after assortment into individual genotypes [75]. Since we have not explicitly modelled the predator species as a third type besides cooperators and defectors, we modulated its saturation by setting a lower uniform rate of predation.

We found, that without group selection, cooperators can only survive if there is predation stress, as otherwise cheaters can easily outcompete cooperators. We compared this trivial result to cases where there was periodical resource scarcity and only a subset of the population could survive. We found that cooperators had the highest chance to survive in case of aggregation-based propagation (with or without dispersion at the new habitat)–and this holds even if there is no predation at all. During aggregation-based propagule formation, cooperators receive indirect benefit from the aggregation, as their chance increases to end up in the dispersing propagule, hence cooperators could colonize significantly more habitats with a better cooperator-cheater ratio in each propagule. Interestingly, aggregation-based dispersion (Fig 4E) is indiscriminately beneficial for cooperators (regardless of predation), indicating that group selection is important, and is best combined with dispersal on arrival to minimize initial competition (that would benefit defectors over cooperators). However, this requires some evolved mechanism on the organism’s part, as microbial spores or propagules usually lack active mechanisms to disperse individual calls on arrival, especially when they are desiccated over long dormancy. There may be such mechanisms, but our results indicate that they are not necessary, as a propagule without immediate dispersal (Fig 4F) can still maintain cooperators in the long run.

It is worth comparing the mechanism of aggregation-based dispersion to Wilson’s group selection model [40,41]. In both models, a preferential (better-than-random) assortment of cooperators into propagules ensures that they can fixate in the population. Both models demonstrate the effect of multilevel selection where altruistic and selfish individuals compete at the lower level while group selection facilitates altruists at the higher level, there are some crucial differences. The structured deme model lacks spatiality (and size-dependent selection), individuals do not replicate while grouped, and it does not specify the mechanism that ensures better-than-random assortment of individuals into groups (aggregates, propagules). Wilson’s model generally demonstrates that any mechanism (partner recognition, adhesive aggregation, kin selection, etc.) can lead to the stable coexistence of types. For a more detailed analysis, see [76]. We emphasize the effect of spatiality: when propagules do not immediately disperse in the new habitat, it becomes less trivial for cooperators to survive (cf. Fig 4E vs. 4F).

We have investigated random fragmentation, which (according to our results) is not sufficient to maintain cooperators in the long run against cheaters. Compared with the case when there is no colonization, cooperators have a benefit against defectors due to local correlations. We emphasize, that the random fragmentation mechanism maintains spatial patterns, but the transferred subpopulation is not selected according to where the cores of cooperative aggregates are. In this regard, it is an intermediate mechanism in our model between random dispersion (which does not keep population structure) and aggregation-based colonization (which fully retains local population structure).

If there is no predation, aggregation-based propagule formation provides cooperators the highest probability of survival. In our model, there is a constant need for microbes to recolonize new habitats due to periodic resource scarcity but without size-dependent selection, there are no direct mechanisms to facilitate cooperation, like in case of predation [57,77], syntrophic symbioses [78,79]; selective partner recognition, choice and retention [80,81]; public goods [82], especially with private benefits [83]; quorum sensing [84,85], especially with honest signalling [86]; nonhomogeneous spatial environment [87] or interaction graph, or other means to increase the (average) reciprocity of altruists [42]. If there is no direct benefit of cell-to-cell association, preferential assortment and group selection means the only chance for cooperators to survive in the long run (Fig 4E and 4F).

There may be three indirect, general benefits for aggregating, besides the direct positive selection due to predation: 1) cells may increase their chance for transmission to the new habitat (if cooperators form propagules that colonize); 2) in the new habitat, they may have a higher initial ratio than defectors; and 3) cooperators may experience higher local cooperator densities in the new habitat (when spatial structure is maintained, cf. Fig 4D, 4E and 4F). The latter is not trivially beneficial, as our results attest Fig 4C: high densities cause faster starvation in aggregate cores. In other cases however, the high density and intact spatial/population structure may be needed to maintain local interactions or to meet a quorum threshold [86]. Nevertheless, cooperators benefit from the increased chance to meet with other cooperators. In general, physical [88] or social viscosity [89] benefits cooperators both spatially and on graphs.

The results support our hypothesis that group selection itself is enough to allow cooperators to survive, and size-dependent selection (predation) is not necessary for aggregation to be beneficial. This does not mean that predation (or equivalent factor selecting for larger size) was not involved in any of the aggregative transitions, or that it could not have been the initial benefit of unicells evolving association mechanisms. For this to be decided, explicit clues of prior predation (or equivalent size-dependent) stress could perhaps be identified in aggregative multicellular cases that are not anymore under predation of unicellular predators (or equivalent stressors).

Our minimal model neglects certain factors which could be influential to cooperator survival, like stress-induced aggregation cues or the diffusion of nutrients. When the propagule reaches a new habitat, defectors spread faster than cooperators, and as a result they starve less frequently than cooperators. Without circulation or other means of effectively importing resources, immobile cooperative aggregations suffer from local starvation [90]. Despite this shortcoming, we believe that our model is sufficient to model the group selection of aggregative strains as it represents a “worst case” scenario: limited nutrient diffusion would give cooperators more benefit, hence if they survive under worst circumstances, they have better chances under less stressed conditions.

It is known that in *Dictyostelium discoideum* there are many processes other than aggregation where extracellular signalization plays a role, like repulsion of each other, sensing the local cell concentration, the hunger and stress signals of the neighbours and estimation of the number of cells in the group [91]. We have intentionally ignored regulating mechanisms, like the paracrine signalization or other gene regulatory networks, studied by recent models [57,92], focusing on a minimal model to investigate the individual effects of group selection and size-dependent selection (predation). Likely such regulating mechanisms are evolved features that were not present when aggregative multicellular organisms have emerged.

We have intentionally simplified events of the resource-poor period to an instantaneous propagule formation. In nature, propagule formation is a complex process where cells start to produce diffusible attractive metabolites (e.g. cAMP), start to move, find each other, aggregate and organize the fruiting body and spores [25]. Even during spore formation, there are many processes which can affect the cooperator-defector ratio in the spore, giving room for various mutants that could cheat at different stages of the cooperative process [25,56].

However, in our model cells grow and interact in a spatial structure. In a future experimental design, there should be a full mix of the lattice. This effect, equivalent to disturbances, which are common in microbial communities [93], would render our model more realistic. With this experiment we could separate the effect of spatiality and the effect of the ratio of cooperators-to-defectors in the propagule. Our presumption is that if there are no persistent associations, defectors would gain more advantage. More experimentation is required to decide this question.

The presence of mutants and other species can also stimulate or inhibit aggregation, e.g. factors from *Escherichia coli* can decrease the success of aggregation of *Dictyostelium* [94]. Here, we have opted not to investigate the effect of mutations and other species to keep the model simple and to focus on intraspecific dynamics. In the future, it would be worth investigating invasion dynamics of mutants (ecotypes or other species), e.g., whether resident cooperators can withstand the invasion of novel cheaters and whether cooperators can invade a defector population. Also, in any realistic situation, extinct defectors can reappear due to mutation or migration (omitted in our model). Adaptive dynamics [95,96], can be used to investigate the evolutionary stability and robustness of cooperators.

We conclude that spatiality and group selection play a significant role in enabling and establishing cooperative group formation during aggregative multicellularity. In our individual based model, cooperators have a higher probability of survival and could colonize more habitats per unit time than cheaters, even if there is no predation at all, solely as the result of non-random propagule formation, respecting spatial structure (cooperative aggregates). Besides spatiality, predation (or equivalent size-dependent selection) also plays an important role when there is no environmental resource fluctuation and propagule formation, because it increases the survival probability of cooperators against opportunistic cheaters. If, however, we exclude direct size-dependent factors benefiting cooperators, like predation, they could only survive if the indirect benefit of spatiality and group selection mechanisms are granted.

## Materials and methods

### Description of the model

We designed an individual-based, spatially explicit and stochastic model of a single-celled organism, potentially a prokaryote. The modelled unicellular organism is haploid, asexual and living a motile, free life. There are two independent cell lineages: cooperators and defectors (or cheaters). We assume, that defectors have already appeared in the population, hence we ignore mutation. Therefore, the two types cannot be transformed into each other (for sake of simplicity, we neglected phenotypic plasticity). Cooperators can associate via expressed adhesive molecules, but expression is costly, hence they grow slower than cheaters who do not invest into production hence cannot associate with other cells. Defectors have an advantage during growth, but they cannot form a fruiting body and propagule without cooperators. The parameters of our model can be seen in Table 1.

We modelled the population on a 100×100 lattice, folded into a torus in order to minimize artifacts caused by boundary effects. Each lattice location can be either occupied by a single cell or empty. The state of lattice location is determined by the type and internal state of the occupying cell, the amount of resource available at the given location and the set of cells in the Moore neighbourhood (8 neighbours). For each location, we monitored the actual cell type, the amount of available resource, the association state of the cell, the energy level of the cell and the number of associated neighbours of the cell. Simulations were started from an average of 200 defector and 200 cooperator cells dispersed randomly over the lattice (unless stated otherwise, e.g. in Fig G in S1 Text). The cells have equal amounts of initial energy *e*_*0*_ and the resource in the habitat is uniformly distributed at the beginning: *r*_*ij*_ = *r*_0_ for each location at the *i*^*th*^ row and *j*^*th*^ column in the lattice.

We used asynchronous lattice update to minimize computational artefacts. During an update, a cell was randomly selected from the lattice. The cell’s internal state was updated in the same order for each update. We measured time in number of updates. The 100×100 lattice is fully updated after on average of 10^4^ individual updates. We have defined a simulation length *T* = 3∙10^7^ (unless stated otherwise) that ensured, on average, the competitive exclusion of one type. After executing update rules, cells meeting elimination conditions are removed. Cells die either when their energy value is reduced to 0, or they are preyed, or another cell in the neighbourhood divides and the offspring overwrites the focal cell or when the cell is excluded from the propagule that is transferred to the new habitat.

Update starts by removing a constant value *c* from the energy level of the cell, representing the energy consumption of the active metabolism. We used *c*_*C*_ = 10 for cooperators and *c*_*D*_ = 8 for defectors. Cells increase their energy level by consuming resources available at the given location homogeneous, growing cells consume resources at their locations. The resource does not diffuse over the lattice and is limited, not being replenished between updates.

Cells reproduce when they reach a given energy threshold *d* = 190 (same for cooperators and defectors). If the amount of energy of a given cell is above *e*, the cell divides (splits) with a constant given probability *S* = 0.7 in a given update if there is empty space in the direction of the division (there is no upper limit of the energy value). If the target location is occupied, then the division will happen with *S*∙*O* where *O* = 0.5 is the probability of overwriting another cell (same for cooperators and defectors). After a successful division, both daughter cells receive half the energy of the parent. The daughter cell at the new location is never associated after the division. Cells inherit the type of their parent (cooperator or defector). We have ignored the effect of mutations.

Cells can associate with each other. There are 2 participants of this process: the initiator and the acceptor cell. The initiator can only be a cooperator, but the receiver can be of either type. The association happens with a given probability *A*. Associated cells can dissociate. The probability of dissociation is the same for the two cell types, *D* (most of the times *D* = 0.3) and *D* is the same for cooperators and defectors (independent of the local or total number of associated neighbours). An initiator or an acceptor cell cannot dissociate in the update of the given cell. Unassociated cells can move actively; however, they can only move into unoccupied locations. Their movement is random, they are able to move into the 8 neighbouring locations with equal probability. With 1/9 probability, an unassociated cell stays where it is. The energy cost for movement is *m* = 8. Associated cells do not move.

We defined environmental periods rich and poor in resources. While the total amount of available resource in the lattice is at or above *R* = 0.4∙10^6^ = 0.4∙*r*_0_ (40% of the total original amount), cells can grow as they consume resources (resource-rich period). If the total amount of available resources goes below *R*, the resource poor period commences. The poor period is not explicitly modelled, we assume that propagule formations and colonization happen immediately without further updates. The selected propagule is repositioned into an empty lattice with resource distributed over all locations so that *r*_*ij*_ = *r*_0_ for each location at *i*,*j* (the new habitat).

During random fragmentation, a continuous 10×10 submatrix representing the fragment is selected randomly from the lattice and put into the new lattice habitat. In case of aggregation-based propagule formation, the propagule is selected as a square matrix centred on the largest aggregate. In the case of refugium, cells were selected randomly from the old habitat and were put in a 10×10 square matrix in the new one. During random dispersion, the cells were randomly chosen and were put in the new habitat while their coordinate did not change in the lattice. In case of random fragmentation, the aggregation pattern was neglected, but randomly selected cells preserved their location when they were transferred individually to the new habitat. In case of random dispersion and refugium, spatiality was largely neglected too, so cooperative aggregation likely had a smaller positive effect on maintaining cooperators.

We used the following parameters for the probability of cell-division (splitting) and the amount of energy required for division: (*S* = 0.7, *e* = 190, respectively, same for cooperators and for defectors). The probability of association *A* and dissociation *D* of cooperators (*C* in index) and defectors (*D* in index) were *A*_*C*_ = 0.95, *A*_*D*_ = 0, *D*_*C*_ = *D*_*D*_ = 0.3. These parameters were denoted with white asterisk in Fig 4. The length of the simulation was *T* = 3∙10^7^ updates, during which the whole lattice was updated approximately 300 times.

**Fig 7:**
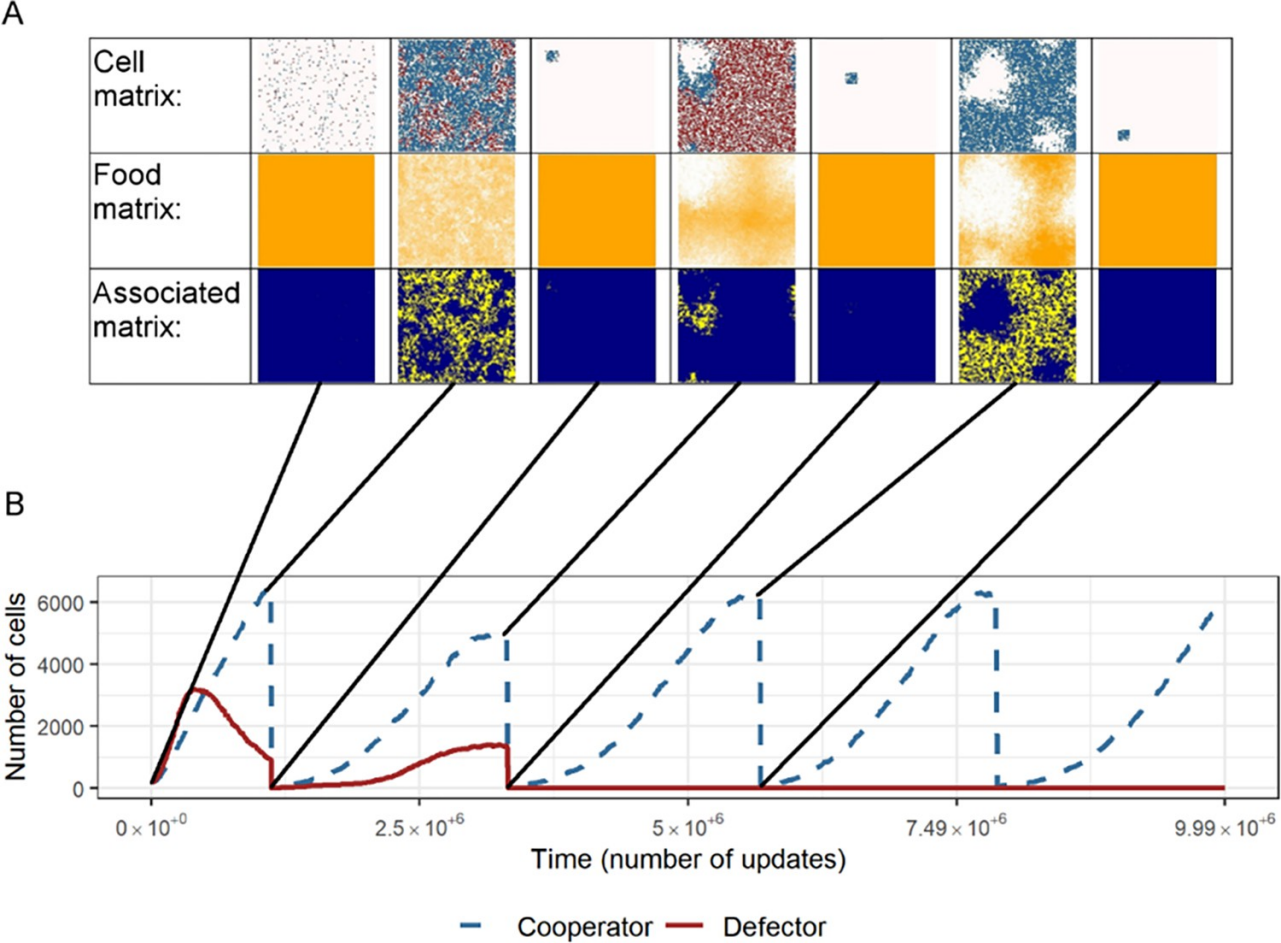
Dynamical changes in the population composition. ***A***: Cell, food and association matrices represent the population at every timestep. Cell matrix row: blue represents cooperators, red defectors, empty cells are white). Food matrix row: orange represents available resource amount, white denotes resource-poor cells. Association matrix row: the brighter a cell is, the more associated neighbours it has (from 0 to 8). ***B***: Temporal dynamics of the population composition. Abrupt abundance drops indicate successive colonization events of new habitats after propagule formation. Note, that in the second colonization, only cooperators were transferred, and defectors were lost.

Fig 6 shows some representative states of the population while Fig 7 shows population dynamics over time with the underlying population, association, and resource lattices.

We investigated the change in relative frequencies of each type over time. Our goal was to find out under what conditions cooperators can survive. The length of each simulation (i.e., number of updates) was defined so that most of the time, only one type survives at the end. Simulations were stopped when there was aggregation-based propagule formation, and a minimal cooperator ratio was set and there was no suitable aggregation for spore formation.

We performed two experiments. In the first experiment, we tested whether spatiality increases the survival probability of cooperators under different mechanisms of (or no) colonization. The second experiment was designed to find out how size dependent selection (predation) affects the survival probability of cooperators in case of the different propagation mechanisms.

### Size-dependent selection (predation)

To model size-dependent selection, we assumed a nondescript microbial predator species that preys on the modelled species unselectively and without saturation. We have modelled predation as a probability of a cell being captured and eaten by the predator at any given timestep.

Predation affects the survival probability of cells. The probability depends on the number of associated neighbours, as follows:

$$P=\frac{1}{{\left(N_{a}+2\right)}^{s}},$$ Eq 1

where *N*_*a*_ is the number of associated neighbours and *s* is the strength of predation (see Fig K in S1 Text).

### Propagule formation and colonization

In order to implement group selection, we designed five types of propagule formation: three types of random and two aggregation-based (see Fig 2). In all cases, the propagule size (number of transferred cells) is *N*_*P*_ = 100 (i.e., 1% of the lattice). In case of mechanisms that represent structural reproduction (fragmentation, propagule formation Fig 2D, 2E and 2F), the propagule is a 10×10 continuous square subpart of the lattice that preserves the spatial pattern of cells in case of D and F but not of E. In case of random dispersion and refugium (Fig 2B and 2C), *N*_*P*_ cells were chosen randomly from the lattice and were either dispersed (B) or transferred into a continuous square (C), in which the spatial pattern did not reflect the previous positions of the cells. The prerequisite of propagule formation is that there are cooperator cells in the lattice (because adhesion is required for the fruiting body formation). If no cooperator remains, there will be no more colonization (the simulation effectively ends). In case of propagule formation, it was always checked whether the propagule contains cells in order to ensure it is not empty. In case of random fragmentation, we made sure that the fragment is not empty. In case of random fragmentation, a propagule could only form if the number of cells is above 10 in order to eliminate random extinctions due to extremely small population sizes. In case of aggregation-based propagation (Fig 2E and 2F), individuals with the most associated neighbours form the centres of aggregates. An individual from these is chosen randomly, forming the centre of the propagule to be transferred to the new habitat.

At the new habitat, the cell matrix, association matrix, and energy matrix are being emptied. The resource matrix is reset to *r*_0_ at each location. The propagule cells are then positioned on the empty lattice, and individuals receive the initial *e*_0_ amount of energy.

## Supporting information

S1 Text Aggregative multicellularity SI 240220.pdf: Supplementary Information containing statistical analyses, and additional figures and tables. All data required to replicate the statistical results and figures are publicly available as comma separated plain text data files at Zenodo: https://doi.org/10.5281/zenodo.10698049. The source code to reproduce simulations and data is publicly available at the following GitHub repository: https://github.com/OszoliI/cooperative-multicellularity-in-an-individual-based-spatial-model.
(PDF)
