# Supplementary material for: Group-selection via aggregative propagule-formation enables cooperative multicellularity in an individual based, spatial model
Source: PLoS Comput Biol. 2024 May 7;20(5):e1012107. doi: 10.1371/journal.pcbi.1012107 (PMC11101088; doi:10.1371/journal.pcbi.1012107)
Supplement: S1 Text — All data required to replicate the statistical results and figures are publicly available as comma separated plain text data files at Zenodo: https://doi.org/10.5281/zenodo.10698049. The source code to reproduce simulations and data is publicly available at the following GitHub repository: https://github.com/OszoliI/cooperative-multicellularity-in-an-individual-based-spatial-model. (PDF) [file pcbi.1012107.s001.pdf]

Group-selection via aggregative propagule-formation enables cooperative multicellularity in an individual based, spatial model

Supplementary Material

István Oszoli[1], István Zachar[1,2*]

[1] Department of Plant Systematics, Ecology and Theoretical Biology, Eötvös Loránd University, 1117 Budapest, Hungary

[2] HUN-REN Institute of Evolution, Centre for Ecological Research, 1121 Konkoly-Thege Miklós út 29-33., Budapest, Hungary

* Corresponding author

e-mail: zachar.istvan@ecolres.hu

1. Statistical analysis of division count

We have determined the number of cell division events (N_d) between successive colonization events. We then compared the N_d for the various colonization mechanisms (A-F) and with and without predation (Figure *A*). In case of no colonization (A), we measured the number of divisions between successive resource replenishment events (i.e. whenever the initial amount of resources in the lattice goes below 40%, it is replenished). For the different mechanism, see Fig 2 of the main text. We have conducted the statistical analysis with R, using the `tidyverse`, `ggpubr`, `gridExtra`, `dunn.test` and `outliers` packages.

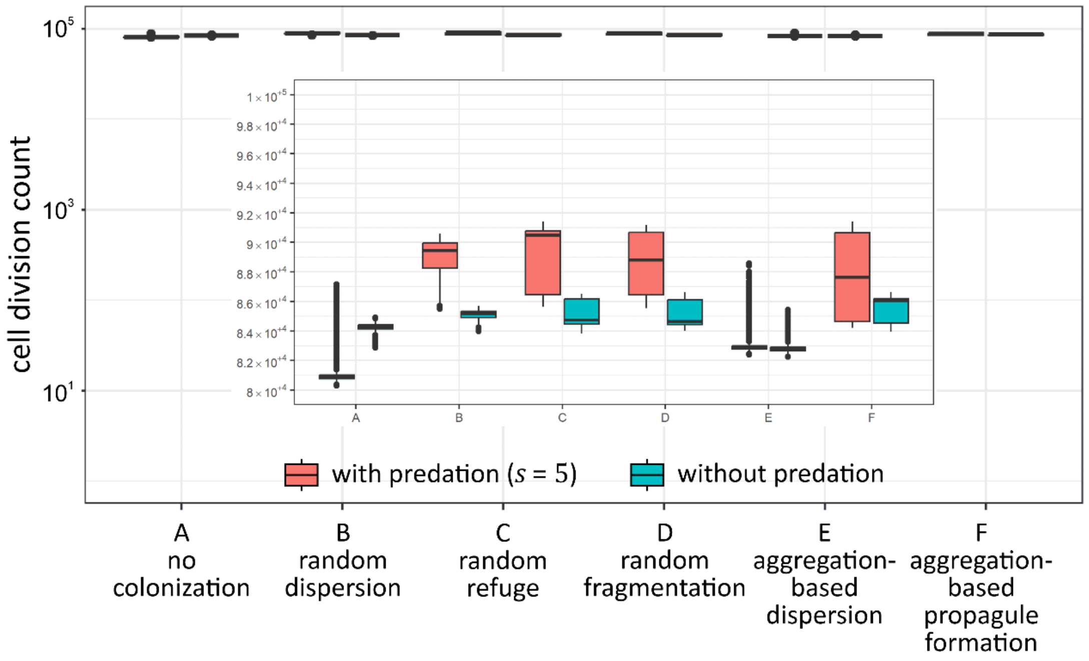

Figure A. Average number of cell divisions in the population between two successive colonization events for the different colonization modes (A-F), with and without predation (size-dependent selection; red and teal boxes, respectively). Each box represents 140 independent simulations. The y axis is on a logarithmic scale. The inset shows the zoomed-in region of interest. Points denote outliers. There is significant difference, though it has practically no effect on the absolute number of cell divisions between the different propagation modes. There is a difference between the cases when predation is or is not in effect, but the difference is still negligible overall. Under predation, cells divide faster on average, because due to the increased mortality, there is always more empty space to divide to. The parameters are as in Table 1 in the main text.

First, we determined whether the data series are normally distributed or not. We have used three methods: asymptotic one-sample Kolmogorov-Smirnov test, Shapiro-Wilk normality test and have visually analyzed the Q-Q plots. In order to perform the Shapiro-Wilk normality test in R, we had to reduce our sample size randomly to 5000 data points. Neither the dataset with, nor the one without predation is normally distributed.

- In the case of the dataset **with no predation**, the results of the asymptotic one-sample Kolmogorov-Smirnov test are: $D = 0.15128$, $p < 2.2 \cdot 10^{-16}$. The results of the Shapiro-Wilk normality test are: $W = 0.90715, p < 2.2 \cdot 10^{-16}$. Figure B.A shows the Q-Q plot.
- In the case of the dataset **with predation**, the results of the asymptotic one-sample Kolmogorov-Smirnov test are: $D = 0.22895$, $p < 2.2 \cdot 10^{-16}$. The results of the Shapiro-Wilk normality test are: $W = 0.82299, p < 2.2 \cdot 10^{-16}$. Figure B.B shows the Q-Q plot.

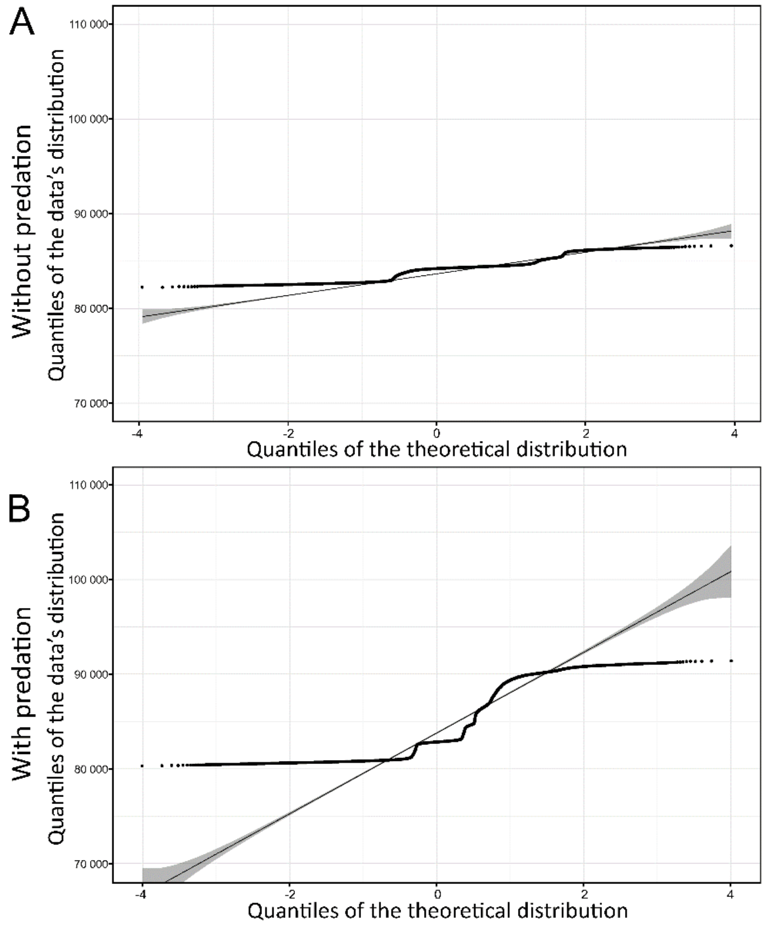

Figure B. Visual analysis of the distribution of the number of cell divisions between successive colonization events plotted against the theoretical normal distribution. The points denote our sample. The straight line with the grey envelope shows where our data points would be if they were normally distributed. ***A****: data without predation,* ***B****: data when predation was in effect.*

Due to the non-normal distribution of our data, we have used Kruskal-Wallis rank sum test to compare whether there is a significant difference between the cases with and without predation to see if predation influences the number of divisions or not. We have compared the datasets of with and without predation, ignoring the background variable (colonization type). We have found significant difference in almost all cases, except the (F, D) pair ($\chi^2 = 927.37, df = 1, p < 2.2 \cdot 10^{-16}$), corresponding with Figure *A*.

To compare the different colonization modes (A-F), we have used Wilcoxon-Mann-Whitney test with continuity correction (Table A) and Dunn test (Table B). In these tests, the effect (or lack of) predation was the background variable that we have ignored.

Table A. P values of comparing the number of cell divisions between two colonization events of different colonization mechanisms (A-F) using Wilcoxon-Mann-Whitney test with continuity correction. Values with grey shading are not significant.

	A	B	C	D	E
**B**	$< 2 \cdot 10^{-16}$	–	–	–	–
**C**	$< 2 \cdot 10^{-16}$	$8.7 \cdot 10^{-11}$	–	–	–
**D**	$< 2 \cdot 10^{-16}$	$< 2 \cdot 10^{-16}$	0.0099	–	–
**E**	0.0071	$< 2 \cdot 10^{-16}$	$< 2 \cdot 10^{-16}$	$< 2 \cdot 10^{-16}$	–
**F**	$< 2 \cdot 10^{-16}$	$< 2 \cdot 10^{-16}$	0.0029	0.7713	$< 2 \cdot 10^{-16}$

We have also compared colonization modes using Dunn's test. The results indicate that in most cases, the difference is significant ($\chi^2 = 14826.0887$, $df = 5$, $p < 2.2 \cdot 10^{-10}$). For detailed comparison of groups, see Table B.

Table B. P values of comparing the number of cell divisions between two colonization events different colonization mechanisms (A-F) using Dunn's test. Values with grey shading are not significant.

	A	B	C	D	E
**B**	$< 2 \cdot 10^{-16}$	–	–	–	–
**C**	$< 2 \cdot 10^{-16}$	0.0034	–	–	–
**D**	$< 2 \cdot 10^{-16}$	$< 2 \cdot 10^{-16}$	0.1648	–	–
**E**	0.0311	$< 2 \cdot 10^{-16}$	$< 2 \cdot 10^{-16}$	$< 2 \cdot 10^{-16}$	–
**F**	$< 2 \cdot 10^{-16}$	$< 2 \cdot 10^{-16}$	0.2749	0.2658	$< 2 \cdot 10^{-16}$

Although the data series differ significantly, there is no difference in order of magnitude between the different colonization mechanisms. In addition, pairs of random refugium and random fragmentation (C, D), random refugium and aggregation-based propagule formation (C, F) and aggregation-based dispersion and propagule formation (E, F) are the most similar (demonstrated by the Wilcoxon-Mann-Whitney and the Dunn tests; in case of Dunn, there was no significant difference between C and D). Random dispersion (B) and random refugium (C) are also similar, although there is some difference. Since there is no significant difference in absolute magnitude, these differences can be disregarded having no effect on our main results.

2. Statistical analysis of colonization times

Besides the number of cell divisions, we have also determined the elapsed time (number of updates) between two successive colonization events (*colonization time*, Δt), for the different colonization mechanisms (A-F), and with/without predation (Figure C). We performed Kruskal-Wallis rank sum test to compare the Δt of the different colonization mechanisms. We performed Wilcoxon-Mann-Whitney test to compare the effect of predation for each colonization mechanism.

In most cases, colonization times significantly differ from each other depending on transfer mechanism (A-F). However, since there is no significant difference in absolute magnitude, these differences can be disregarded having no effect on our main results. In case of no colonization (A), we have measured the elapsed time between two successive resource-replenishment events (occurring when the amount of food in the lattice decreases below 40% of the initial food quantity).

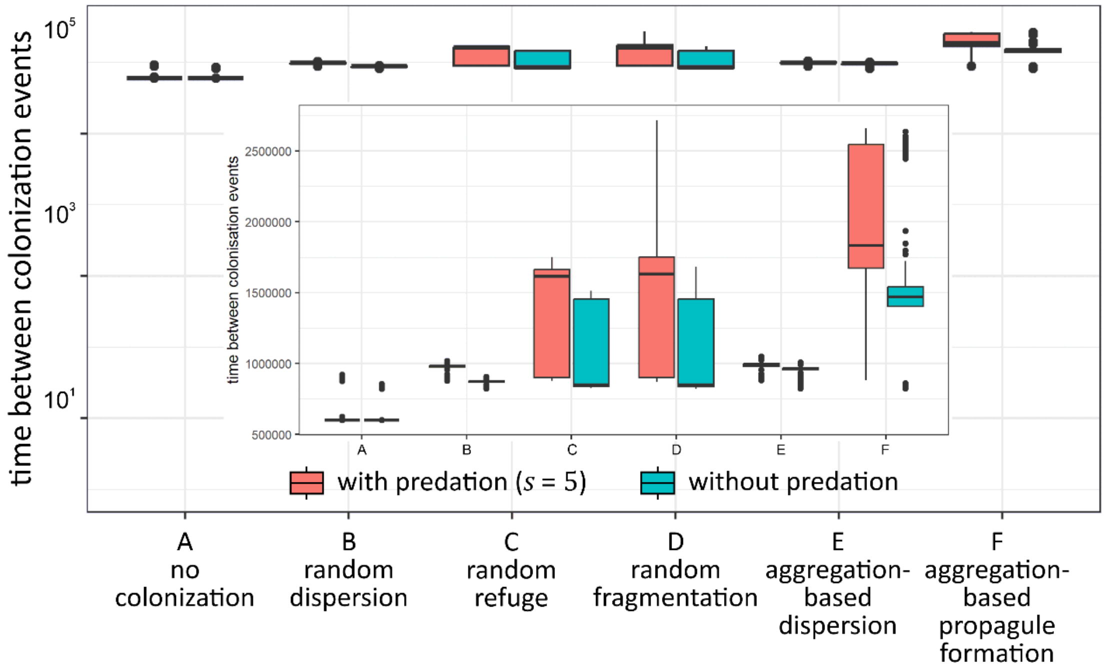

Figure C. Average time elapsed between successive colonization events for the different colonization mechanisms (A-F), with and without predation (size-dependent selection; red and teal boxes, respectively). Each box represents 140 independent simulations. The parameters are as in Table 1 in the main text.

Similarly as before, we have tested whether the data is normally distributed or not. We have used three methods: asymptotic one-sample Kolmogorov-Smirnov test, Shapiro-Wilk normality test and have visually analyzed the Q-Q plots. In order to perform the Shapiro-Wilk normality test in R, we had to reduce our sample size randomly to 5000 data points. Neither the dataset with, nor the one without predation is normally distributed.

- In the case of the dataset **with no predation**, the results of the asymptotic one-sample Kolmogorov-Smirnov test are: $D = 0.2829$, $p < 2.2 \cdot 10^{-16}$. The results of the Shapiro-Wilk normality test are: $W = 0.6722$, $p < 2.2 \cdot 10^{-16}$. Figure D.A shows the Q-Q plot.
- In the case of the dataset **with predation**, the results of the asymptotic one-sample Kolmogorov-Smirnov test are: $D = 0.31574$, $p < 2.2 \cdot 10^{-16}$. The results of the Shapiro-Wilk normality test are: $W = 0.66742$, $p < 2.2 \cdot 10^{-16}$. Figure D.B shows the Q-Q plot.

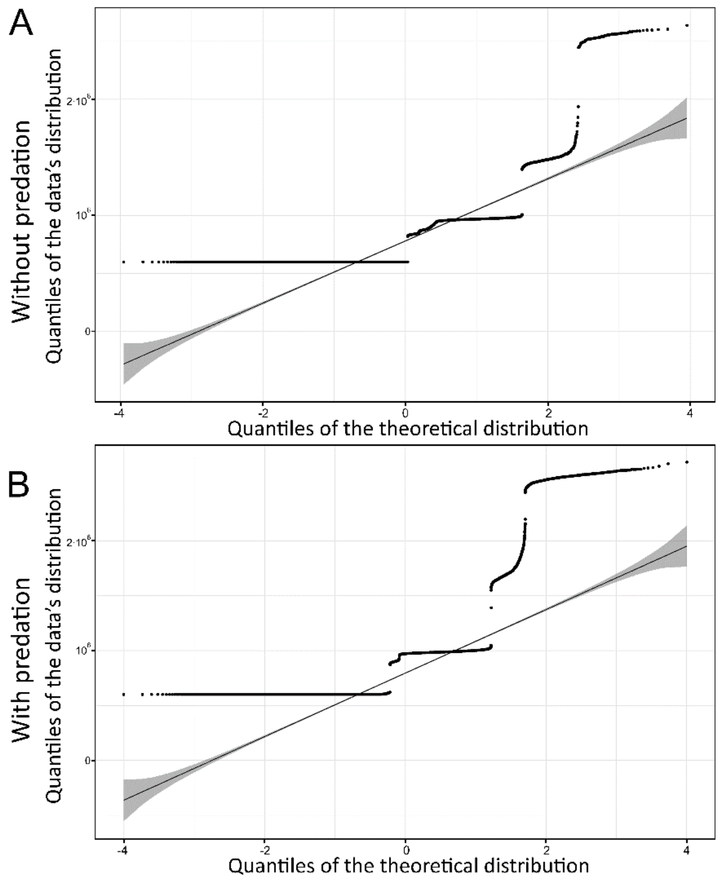

Figure D. Visual analysis of the distribution of the number of cell divisions between successive colonization events plotted against the theoretical normal distribution. The points denote our sample. The straight line with the grey envelope shows where our data points would be if they were normally distributed. ***A****: data without predation,* ***B****: data when predation was in effect.*

Due to the non-normal distribution of our data, we have used Kruskal-Wallis rank sum test to compare whether there is a significant difference between the cases with and without predation to see if predation influences the number of divisions or not. We have compared the datasets with and without predation, ignoring the background variable (colonization type). Results indicate that the mechanisms significantly differ from each other ($\chi^2 = 5431.37, df = 1, p < 2.2 \cdot 10^{-16}$).

To compare the different colonization modes (A-F), we have used Wilcoxon-Mann-Whitney test with continuity correction (Table C).

Table C. Results (p values) of the Wilcoxon-Mann-Whitney test, comparing the colonization times for the different colonization mechanisms (A-F). Cells with grey shading indicate insignificant values. Otherwise, the test indicates that most colonization mechanisms produce different colonization times.

	**A**	**B**	**C**	**D**	**E**
**B**	$< 2 \cdot 10^{-16}$	–	–	–	–
**C**	$< 2 \cdot 10^{-16}$	$< 3.1 \cdot 10^{-10}$	–	–	–
**D**	$< 2 \cdot 10^{-16}$	$< 3.6 \cdot 10^{-9}$	0.2009	–	–
**E**	$< 2 \cdot 10^{-16}$	$< 2 \cdot 10^{-16}$	0.0065	0.0205	–
**F**	$< 2 \cdot 10^{-16}$	$< 2 \cdot 10^{-16}$	$< 2 \cdot 10^{-16}$	$< 2 \cdot 10^{-16}$	$< 2 \cdot 10^{-16}$

We have also compared colonization modes using Dunn's test ($\chi^2 = 22917.4949$, $df = 5$, $p = 0$), see Table D.

Table D. Results (p values) of Dunn's test for comparing the colonization times for the different colonization mechanisms (A-F). Cells with grey shading indicate non-significant values. Dunn's test: $\chi^2 = 22917.4949$, $df = 5$, $p = 0$.

	A	B	C	D	E
**B**	$< 2 \cdot 10^{-16}$	–	–	–	–
**C**	$< 2 \cdot 10^{-16}$	0.0006	–	–	–
**D**	$< 2 \cdot 10^{-16}$	0.0003	0.4415	–	–
**E**	$< 2 \cdot 10^{-16}$	$< 2 \cdot 10^{-16}$	0.0829	0.0570	–
**F**	$< 2 \cdot 10^{-16}$	$< 2 \cdot 10^{-16}$	0.2749	$< 2 \cdot 10^{-16}$	$< 2 \cdot 10^{-16}$

While data series may differ significantly, there is no difference in order of magnitude between the different colonization mechanisms. Regarding the elapsed time between colonization events, random refugium differs mostly from the rest. Since there is no significant difference in absolute magnitude, these differences can be disregarded having no effect on our main results.

3. Supplementary table and figures

Table E. The life cycles emerged in the computer simulations of [1]*, according to model parameters.*

**Parameter values**	**Life cycle**
high k, low s<br>variable s, $k \geq 3$	Multicellular only at environment 1
high k, high s	Oscillating only in environment 1, groups frequently fall apart
low k, high s<br>variable s, $k = 3$	Constitutively multicellular.
s equals to the lowest values of k<br>variable s, $k = 3$	Multicellular in all environments, but groups fall apart during the transition from environment 1 to environment 2
low k, high s	Multicellular in all environments, but groups fall apart during the transition from environment 2 to environment 1
intermediate k equals to s	More life cycles live with each other
low k, high s	Multicellular in all environments, but groups fall apart during all type of environment transitions

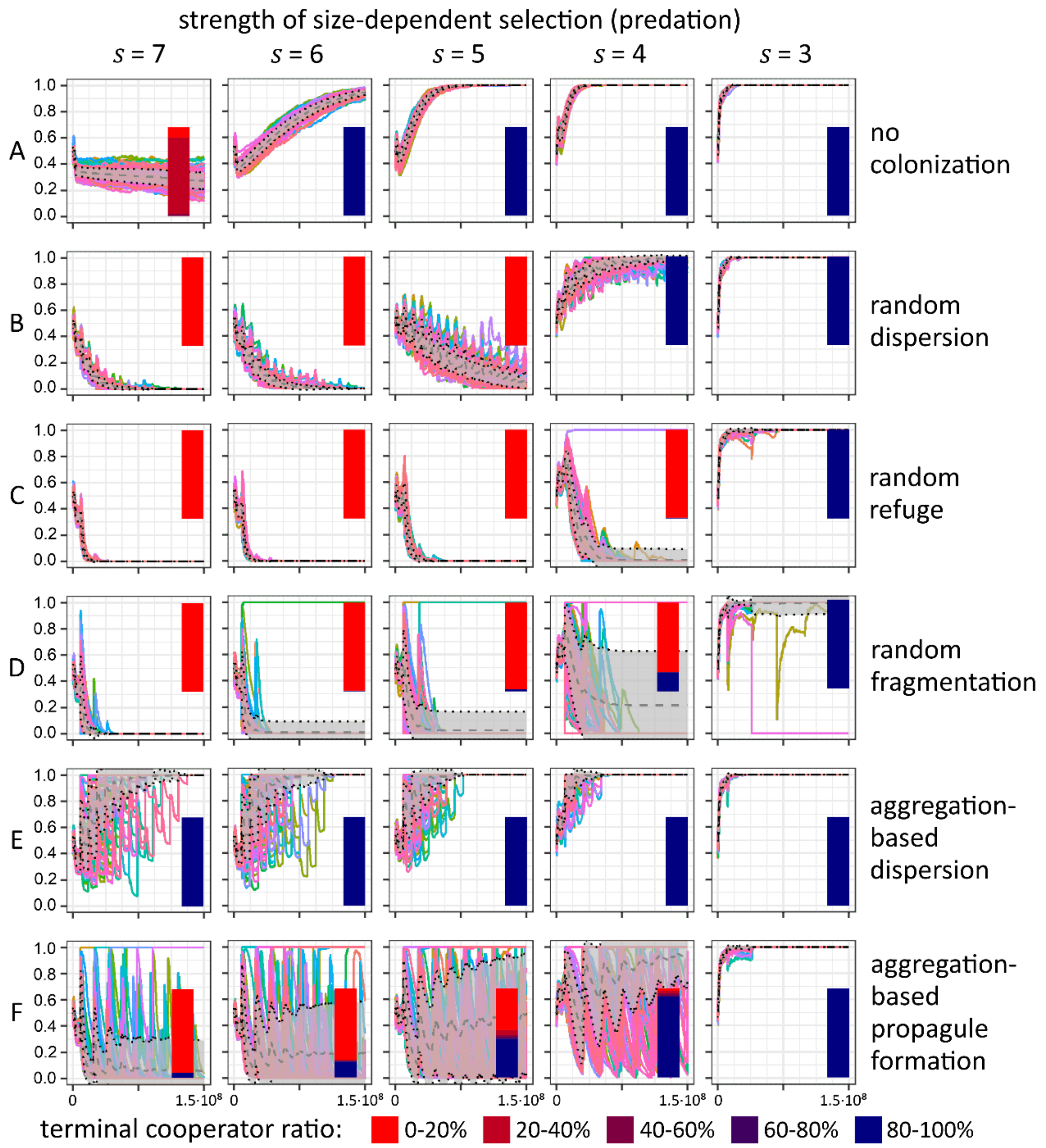

Figure E. The effect of size-dependent selection strength on cooperator survival. Individual plots show the change in the relative frequency of cooperators over time according to the strength of size dependent selection (predation; columns) and propagation mechanism (rows). Each plot shows the time-evolution of 140 independent simulations (coloured lines). The dashed line represents the mean of the relative frequency of cooperators over all simulations, the dotted lines enveloping the grey area represent the mean +/- standard deviation over all 140 simulations. The bar charts give a visual cue of the distribution of independent simulations, showing the terminal ratio of cooperators of individual trajectories categorized into five discrete groups (see legend). Figure F shows only the bars, with scales, for better comparison. The selection strength increases nonlinearly with decreasing s *to the right (as per Eq. 1 in main text, see Figure K). As the strength of predation decreases, the survival probability of cooperators decreases too, as defectors are less and less stressed by predators. This effect is most pronounced in the case of aggregation-based propagule formation, where increasing predation quickly gives an advantage to cooperators. The parameters are as in Table 1 in the main text, with the following difference:* $T = 150\,000\,000$.

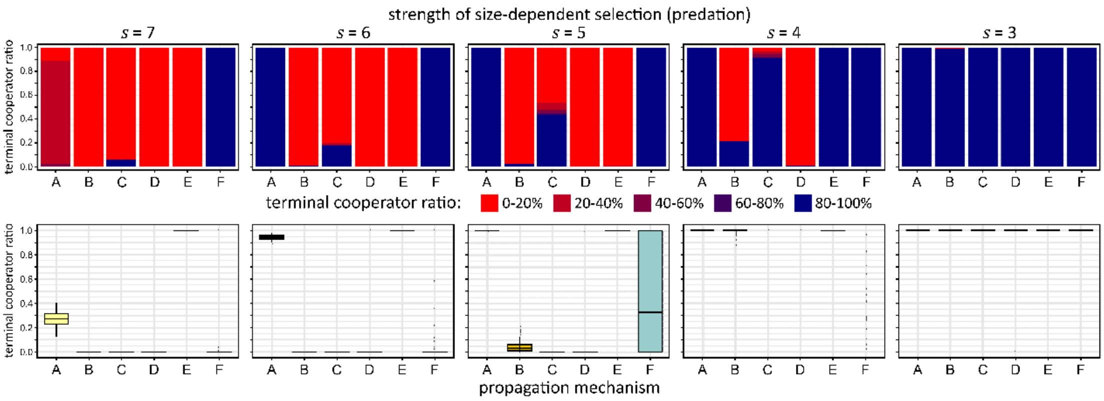

*Figure F. Relative frequency of cooperators in the last generation of the simulations of Figure E, depending on propagation mechanism (A-F) and predation strength s (columns, increasing to the right). The y axis denotes the relative frequency of cooperators in the last generation. Top row: Terminal frequencies are categorized into five bins, as per the legend. Bottom row: Box plots show the distribution of terminal cooperator frequencies. Propagation mechanisms are: **A**: no colonization, **B**: random dispersion, **C**: random refuge, **D**: random fragmentation, **E**: aggregation-based dispersion, **F**: aggregation-based propagule formation. The parameters are in Table 1 in the main text, with the following difference: $T = 150\,000\,000$.*

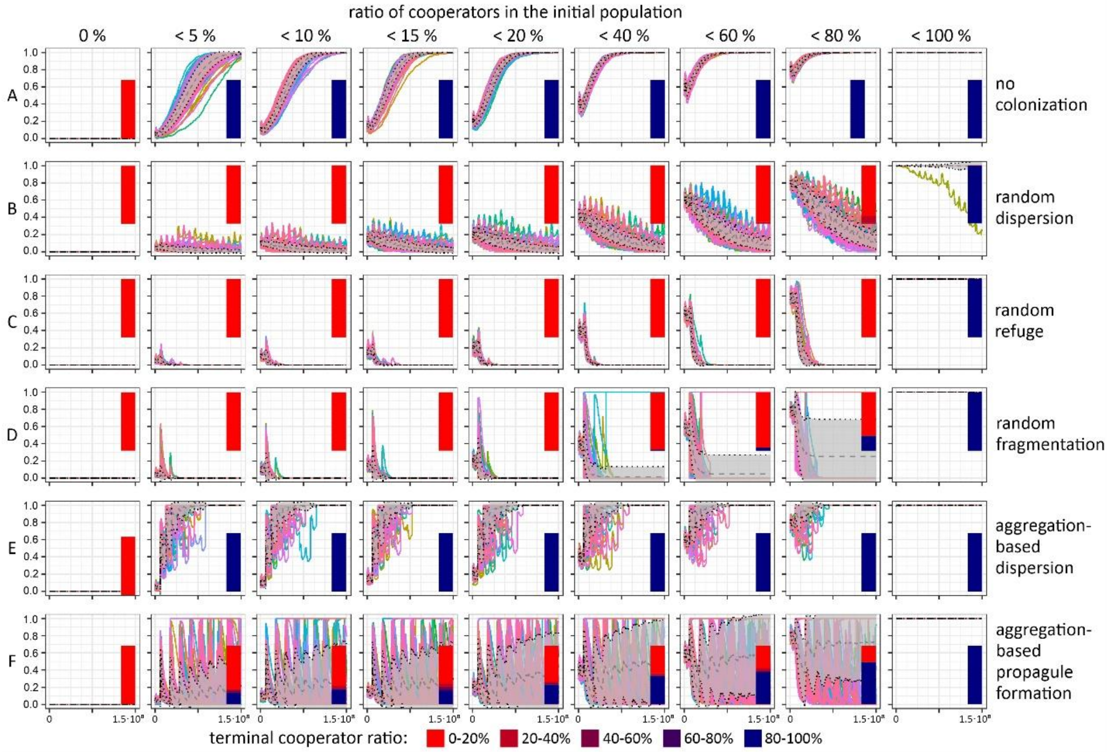

Figure G. The effect of initial cooperator ratio on cooperator survival. Individual plots show the change in the relative frequency of cooperators over time, under predation stress, according to the initial ratio of cooperators (columns; note the nonlinear scaling of values) and propagation mechanism (rows A-F, see Figure 2 in main text for details). Predation is in effect ($s = 5$). Each plot shows 140 independent simulations (coloured lines). The dashed line represents the mean, dotted lines enveloping the grey area represent the mean +/- standard deviation over all 140 simulations. The bar charts give a visual cue of the distribution of independent simulations, showing the terminal ratio of cooperators of individual trajectories categorized into five discrete groups. Figure H shows only the bar charts with scales, for better comparison. The initial cooperator ratio increases to the right. As the initial ratio of cooperators increases, their survival probability increases too in all cases of propagation. Only in case of random fragmentation and aggregation-based propagule formation manifests a considerable increase in the survival probability of cooperators, indicating that in every other case, the threshold for cooperator survival is either the minimum value (at least a single cooperator initially) or that there should be no defectors at all (100% cooperator). Note, that the last column shows <100%, as the initial population contains a few defectors. The parameters are as in Table 1 in the main text, with the following difference: $T = 150\,000\,000$.

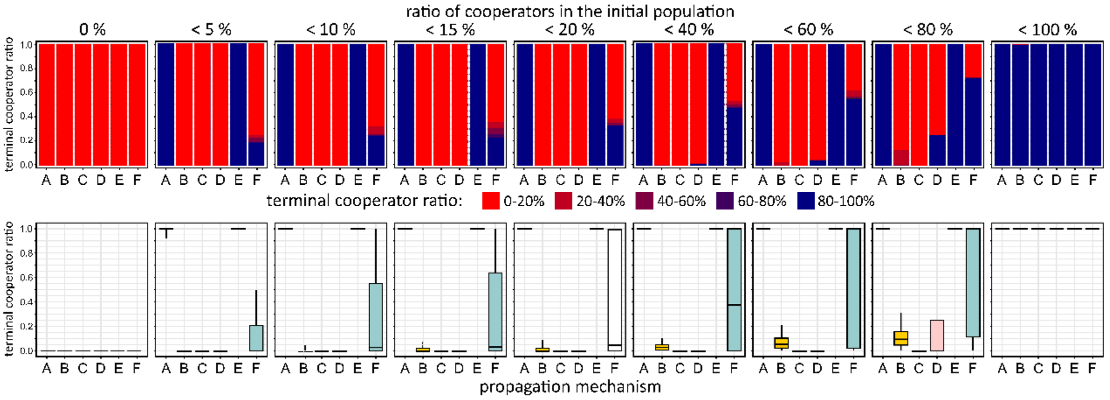

Figure H. Relative frequency of cooperators in the last generation of the simulations of Figure G, depending on propagation mechanism (A-F, see Fig. 2 in main text for details) and the initial ratio of cooperators (columns; note the nonlinear scaling of values). The y axis denotes the relative frequency of cooperators in the last generation. Top row: Terminal frequencies are categorized into five bins, as per the legend. Bottom row: Box plots show the distribution of terminal cooperator frequencies. The parameters are in Table 1 in the main text, with the following difference: $T = 150\ 000\ 000$.

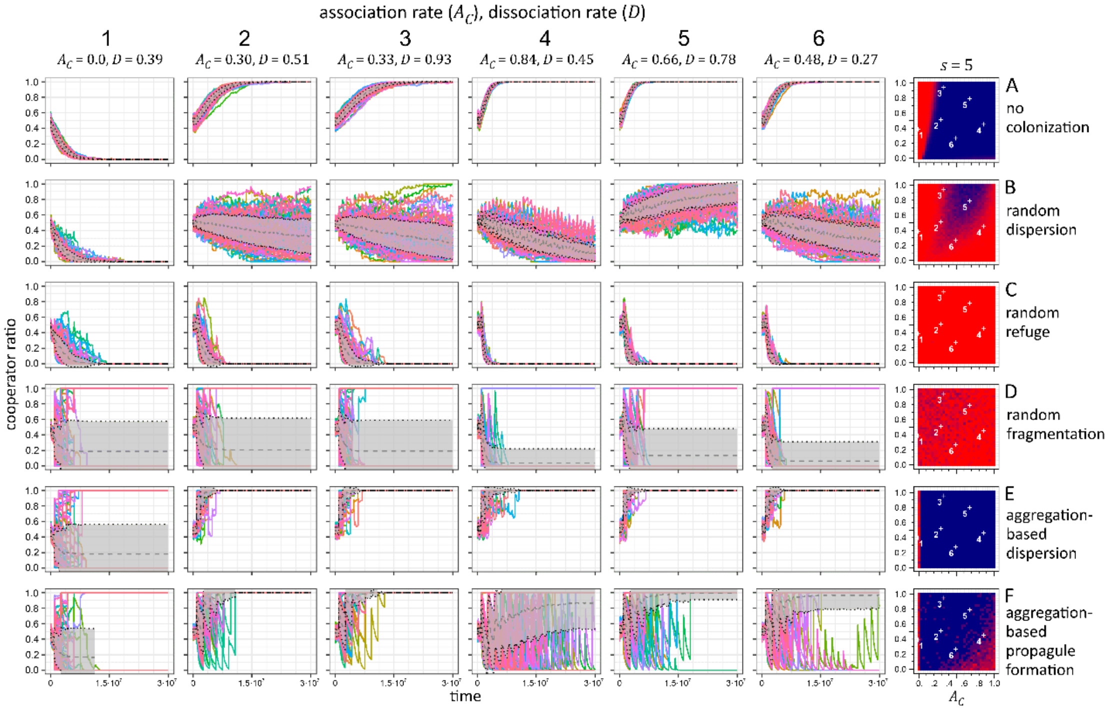

Figure I. Relative frequency of cooperators at selected association and dissociation rates. Predation was in effect ($s = 5$). The six columns (1-6) correspond to specific association-dissociation rate pairs (A_C, D, respectively). Rows A-F correspond to colonization mechanisms (see Fig. 2 in main text for details). Inset maps at the right reproduce the respective plots of Fig. 4 of the main text indicating the positions of the numbered (A_C, D) pairs.

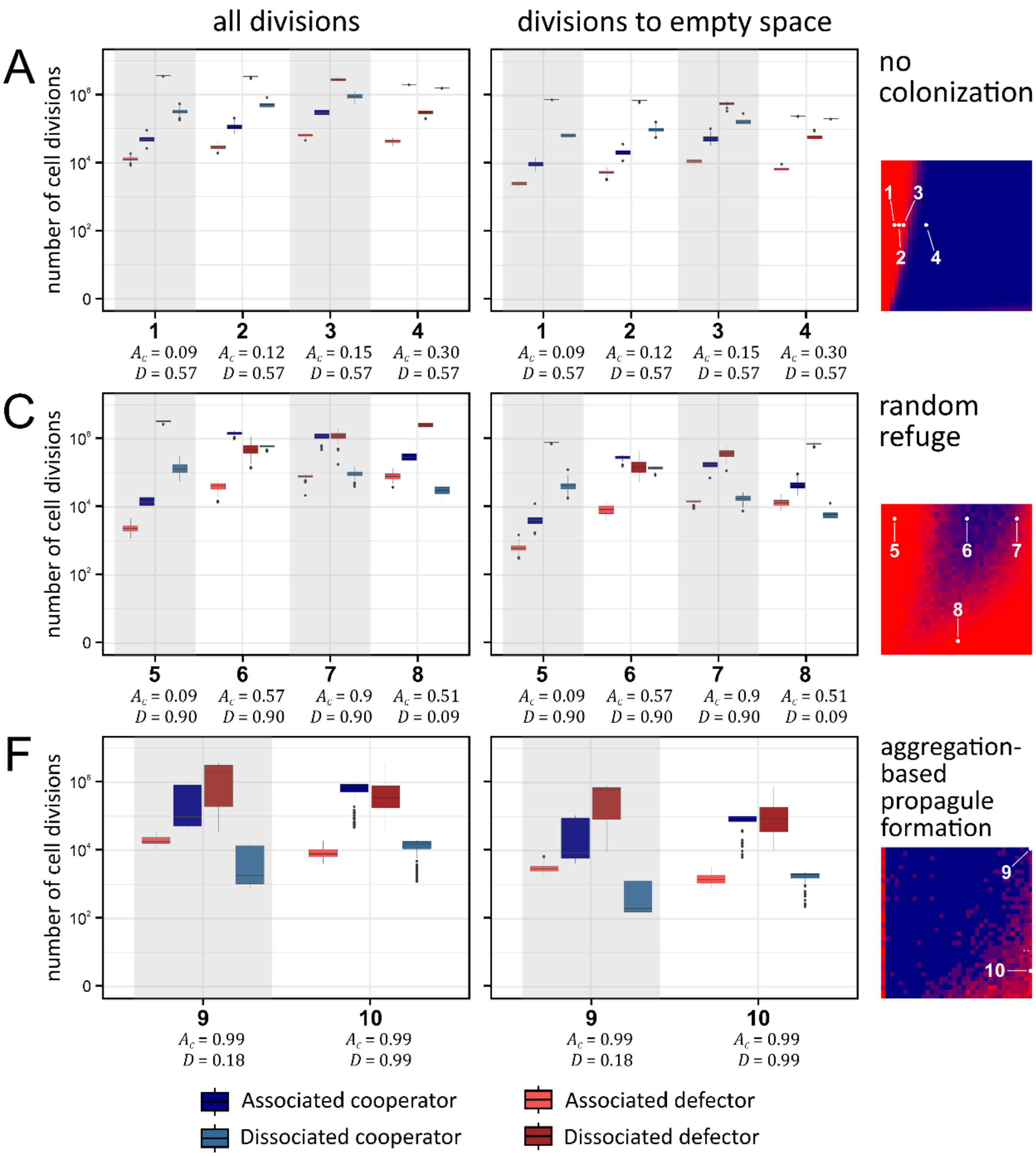

*Figure J. The total number of cell divisions in case of selected propagation mechanisms and association/dissociation pairs (A_C, D), categorized by the cell type (bluish cooperator, reddish defector). Only those propagation mechanisms are shown, where there was an interesting pattern to explain in the respective heatmap of Fig. 4 of the main text (right). The coordinates for various association/dissociation probability pairs (x axis) are indicated by indexed points overlaid on the respective heatmaps. Predation was in effect ($s = 5$). The y axis represents the number of cell divisions. In the left column, all divisions are counted; in the right column, only those divisions are counted where the daughter cell occupied an empty space. Note, that in most cases, the boxes corresponding to dissociated cooperators and associated defectors are too thin to see their color. In case of **no colonization** (**A**), the advantage of cooperators is the result of predation stress strongly affecting defector mortality. As the association probability increases, the survival (and division) probability of cooperators also increases. Cooperators at smaller association probabilities mostly divide when dissociated (**A1, A2, A3**). Increasing the association rate above a threshold causes associated cooperators to divide more than dissociated ones, because most cooperators are now associated (**A4**). In case of **random refugium** (**C**), the benefit of association is negligible at a low association probability (**C5**), and only dissociated cells divide. Since defectors are better competitors when free, they divide more and ultimately win over cooperators. At medium association and high dissociation rates (**C6**), associated cooperators divide more, often leaving the aggregate, and the resulting dissociated cooperators will further divide, winning over defectors (**C7**). However, if the dissociation rate is smaller than the association rate, cooperators cannot leave the aggregate, hindering effective division in dense aggregates (**C7, C8**). In case of **aggregation-based propagule formation** (**F**), at low dissociation rates (**F9**) defectors easily associate at the edge of aggregates, preventing neighbouring cooperators to effectively divide. If the dissociation probability is high enough, cells more easily leave aggregates and thus can divide into empty space more often (**F10**). This is more pronounced, as there is predation and defectors at the edge die more frequently.*

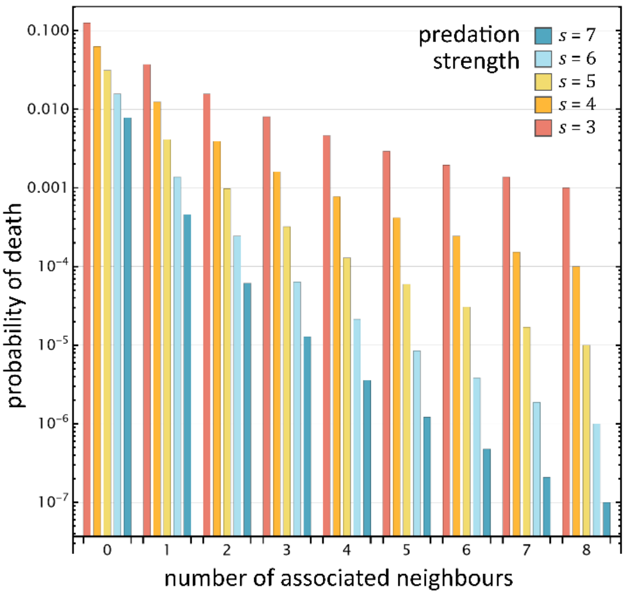

Figure K. The probability of death from size dependent selection (predation) depending on the number of associated neighbours (x axis) and the strength of predation s (differently colored bars), according to Eq. 1 of the main text. Note the logarithmic y scale. The smaller s is, the higher the selection strength will be.

4. Reference

1. Staps M, Gestel J van, Tarnita CE. Emergence of diverse life cycles and life histories at the origin of multicellularity. Nature Ecology & Evolution. 2019 Jul;3(8):1197–1205.
